# *Mycobacterium abscessus*-Induced Granuloma Formation Is Strictly Dependent on TNF Signaling and Neutrophil Trafficking

**DOI:** 10.1371/journal.ppat.1005986

**Published:** 2016-11-02

**Authors:** Audrey Bernut, Mai Nguyen-Chi, Iman Halloum, Jean-Louis Herrmann, Georges Lutfalla, Laurent Kremer

**Affiliations:** 1 Centre d’études d’agents Pathogènes et Biotechnologies pour la Santé, FR3689, CNRS, Univ Montpellier, Montpellier, France; 2 DIMNP, CNRS, Univ Montpellier, Montpellier, France; 3 UMR1173, INSERM, Université de Versailles St Quentin, Montigny le Bretonneux, France; 4 INSERM, CPBS, Montpellier, France; McGill University Health Centre, CANADA

## Abstract

*Mycobacterium abscessus* is considered the most common respiratory pathogen among the rapidly growing non-tuberculous mycobacteria. Infections with *M*. *abscessus* are increasingly found in patients with chronic lung diseases, especially cystic fibrosis, and are often refractory to antibiotic therapy. *M*. *abscessus* has two morphotypes with distinct effects on host cells and biological responses. The smooth (S) variant is recognized as the initial airway colonizer while the rough (R) is known to be a potent inflammatory inducer associated with invasive disease, but the underlying immunopathological mechanisms of the infection remain unsolved. We conducted a comparative stepwise dissection of the inflammatory response in S and R pathogenesis by monitoring infected transparent zebrafish embryos. Loss of TNFR1 function resulted in increased mortality with both variants, and was associated with unrestricted intramacrophage bacterial growth and decreased bactericidal activity. The use of transgenic zebrafish lines harboring fluorescent macrophages and neutrophils revealed that neutrophils, like macrophages, interact with *M*. *abscessus* at the initial infection sites. Impaired TNF signaling disrupted the IL8-dependent neutrophil mobilization, and the defect in neutrophil trafficking led to the formation of aberrant granulomas, extensive mycobacterial cording, unrestricted bacterial growth and subsequent larval death. Our findings emphasize the central role of neutrophils for the establishment and maintenance of the protective *M*. *abscessus* granulomas. These results also suggest that the TNF/IL8 inflammatory axis is necessary for protective immunity against *M*. *abscessus* and may be of clinical relevance to explain why immunosuppressive TNF therapy leads to the exacerbation of *M*. *abscessus* infections.

## Author Summary

The incidence of non-tuberculous mycobacterial infections has recently increased and has even surpassed tuberculosis as a public health concern in many developed countries. These infections require long treatment regimens that are often unsuccessful. Among these, *Mycobacterium abscessus* has emerged as perhaps the most difficult-to-manage pathogen, especially in cystic fibrosis patients. Unfortunately, very little is known regarding the contributions of the pro-inflammatory and innate immune responses during *M*. *abscessus* infection. Here, we exploited the transparency of zebrafish embryos to study, at high resolution, the interactions of *M*. *abscessus* with macrophages and neutrophils, and found that both cell types are required to control the infection. We also describe the dramatic consequences of impaired TNF/IL8 immunity on the outcome of the infection. Most importantly, by tracking the dynamics of neutrophil mobilization, we demonstrated the crucial role of these cells in the formation and integrity of protective granulomas. Together, our data provide a significant advance in deciphering the immunopathology of *M*. *abscessus* infection, which is particularly relevant for understanding the exquisite vulnerability of cystic fibrosis patients to this bacterium.

## Introduction

The rapidly-growing non-tuberculous mycobacteria (NTM), *Mycobacterium abscessus* (*Mabs*), is an emerging pathogen that causes a wide clinical spectrum of syndromes, including skin and soft tissues infections and pseudotuberculous pulmonary infections, especially in patients with underlying lung disorders [1–3]. *Mabs* is considered to be the most pathogenic NTM affecting cystic fibrosis (CF) patients, and is often associated with a dramatic decline in lung function and even death in these patients [4]. This organism is notorious for being intrinsically resistant to most antibiotics and disinfectants [5,6,7] and unsuccessful eradication of *Mabs* is a contraindication to lung transplantation in many CF centers, leaving patients without any therapeutic option. Despite being a rapid grower, *Mabs* can persist for years or decades within the lungs of infected patients [8,9] where it forms organized granulomas [10]. These hallmarks of pathogenic mycobacteria are composed of infected macrophages surrounded by additional macrophages, neutrophils and lymphocytes, and the centers of these tightly aggregated structures can develop caseous necrosis [10]. The pathways leading to *Mabs* granuloma formation and maintenance, however, have been poorly characterized.

*Mabs* exhibit rough (R) and smooth (S) morphotypes that, as a consequence of alterations within the GPL biosynthetic/transport gene cluster, differ in the amounts of surface-associated glycopeptidolipids (GPL) [11]. Pulmonary *Mabs* infections in CF patients that are characterized by chronic airway colonization and poor outcomes have been linked to a genetic conversion allowing the S variant to become R [8,12,13]. This is also supported by results in mice and in cultured macrophages, emphasizing the hyper-virulent phenotype of the R compared to the S form [14–16]. MmpL4b is involved in translocation of GPL to the bacterial surface and its absence correlates with the lack of GPL and the highly virulent phenotype of the R variant [11,17,18]. A plausible explanation for the enhanced virulence of the *Mabs* R morphotype is that loss of GPL unmasks cell wall inflammatory-provoking lipoproteins and/or phosphatidyl-*myo*-inositol mannosides known to be TLR2 agonists [19,20]. Despite the demonstration that *Mabs* R induces a stronger TLR2-mediated TNF response than *Mabs* S [15,20], there is little information regarding the events leading to the inflammatory response in *Mabs* infection and how the inflammation impacts on the outcome of the disease.

By exploiting the optical transparency of the zebrafish embryo model, we confirmed the hyper-virulence of *Mabs* R and revealed the ability of both *Mabs* morphotypes to induce granulomas [17]. The increased virulence of the R variant correlated with a massive production of extracellular serpentine cords of bacteria which, due to their size, prevent phagocytosis, thus suggesting cording as a mechanism of immune evasion. Cords also initiated abscess formation, particularly in the central nervous system (CNS) of the infected animal, with subsequent tissue damage presumably caused by the induction of a potent inflammatory response. Lung injury in CF patients is caused by an intense and persistent pulmonary infection associated with a massive influx of neutrophils into the airways [21]. Thus, scrutinizing the inflammatory and neutrophilic response to *Mabs* infection could provide important advances in our understanding of the *Mabs* immunopathology and the unexplained innate susceptibility of CF patients to these infections.

Herein, through the use of loss- and gain-of-function approaches coupled with fluorescent reporter zebrafish lines and high resolution imaging, we have dissected the TNF/IL8-mediated signaling pathway that contributes to immuno-protection against *Mabs* infection. Our findings unraveled the crucial and dual role of TNF in activating the macrophage bactericidal activity, in restricting intracellular bacterial growth and, importantly, in neutrophil recruitment for the generation and maintenance of protective granulomas.

## Results

### Macrophages are the primary source of TNF-α during *M*. *abscessus* infection

**Fig 1:**
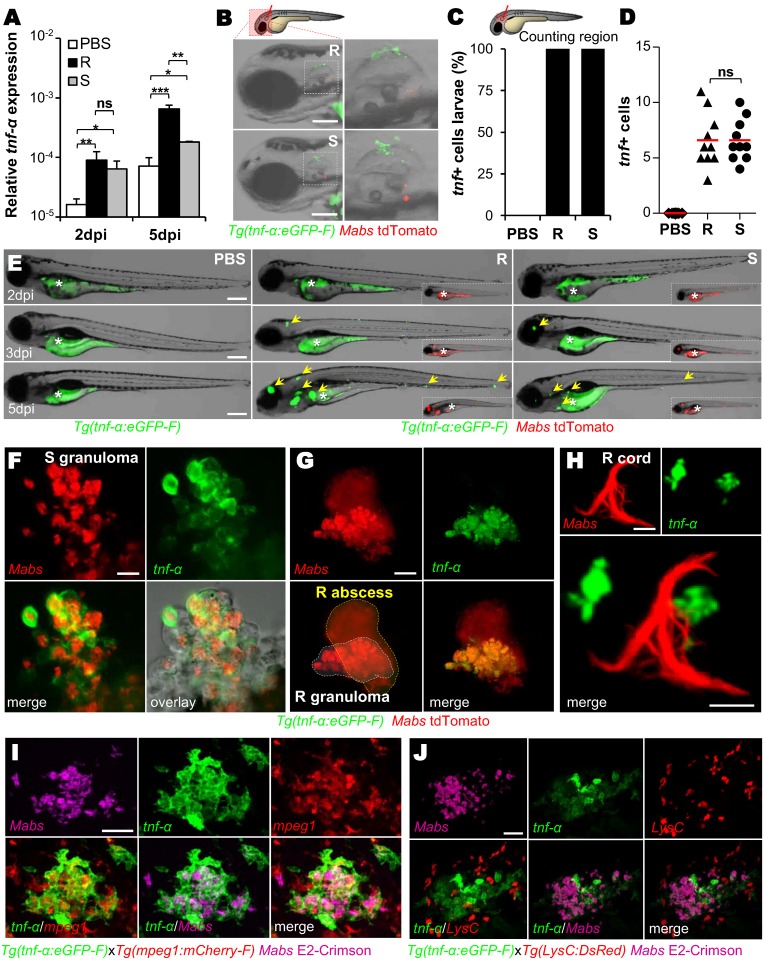
Expression of TNF by macrophages during *Mabs* infection. (A) Measurement of relative *tnf-α* expression by qRT-PCR using *ef1α* as a reference gene in whole embryo over PBS. Around 150 *Mabs* R or S variants were *iv* injected and assessed at 2 and 5 dpi. Mean log_10_ value of three independent experiments with error bars representing the standard error of the mean (SEM). (B-D) *Tg*(*tnf-α*:*eGFP-F*) larvae were injected in the otic cavity with ≈100 *Mabs* R or S variants (tdTomato) or with PBS. (B) Bright-field and fluorescence overlay microscopy showing the representative expression of *tnf* close to the injection site at 2 hpi. Scale bars, 100 μm. (C-D) Mean proportion of infected larvae with *tnf* ^+^ cells (n = 10) (C) and quantification of *tnf* ^+^ cells per infected larvae (each symbol represents individual embryos and horizontal lines indicate the mean values) (D) in (C) after 2 hpi. The data are representative of two experiments. (E) Distribution of *tnfα*-expressing cells revealed by the *tnf-α*:*eGFP-F* reporter transgene (arrows) in whole *Tg*(*tnf-α*:*eGFP-F*) larvae imaged live at the indicated time points post *iv* injection of PBS, *Mabs* R or S (tdTomato, ≈150 colony forming units (CFU)). The yolk (*) is auto-fluorescent. Scale bars, 200 μm. (F-H) Confocal images showing the representative *tnf* expression in a 3 dpi-granuloma (F) (scale bar, 20 μm), in a 5 dpi-brain abscess (G) (scale bar, 50 μm) or close to a 3 dpi-cord (H) (scale bars, 20 μm) in *Tg(tnf-α*:*eGFP-F)* embryos *iv* infected with *Mabs* R (tdTomato). (I-J) Confocal microscopy of a 3 dpi-granuloma showing the *tnf* expression in *Tg*(*tnf-α*:*eGFP-F/mpeg1*:*mCherryF*) (I) or *Tg*(*tnf-α*:*eGFP-F/LysC*:*DsRed*) (J) double transgenic embryos *iv* infected with *Mabs* R (E2-Crimson). Scale bars, 50 μm. Statistical significance was determined by Kruskal-Wallis test with Dunns post-test (A), Fisher’s exact test of a contingency table (C) or one-tailed unpaired Student’s t test (D).

To identify key effectors of the *Mabs*-induced inflammation, the pro-inflammatory profile in R- and S-systemic infected zebrafish embryos was determined and compared at various time points post-infection. Quantitative RT-PCR revealed an induction of *tnf-α* (Fig 1A), *il1-β* and *ifn-γ2* (S1 Fig). Expression of *tnf-α* is induced by both variants at 2 days post-infection (dpi), corresponding to the early appearance of granulomas, and prior to abscess formation [17]. This response was further increased at 5 dpi, with higher levels for R, consistent with the increased TNF release in murine macrophages [15]. This indicates that *Mabs*, notably the R variant, induces a robust TNF response in zebrafish.

*Tg*(*tnfα*:*eGFP-F*) zebrafish larvae, which express farnesylated eGFP under the control of the *tnf-α* promoter [22], were infected with *Mabs* to investigate the nature and the spatiotemporal distribution of the TNF-producing cells. While the control PBS injection in the otic cavity failed to induce eGFP expression, all animals injected with *Mabs* exhibited green fluorescent cells that were recruited and clustered around the injection site as early as 2 hours post-infection (hpi) (Fig 1B and 1C), with equal numbers of eGFP-positive cells recruited in response to S and R variants (Fig 1D). In intravenously (*iv*) infected embryos, eGFP expression was detected from the earliest hours post-infection (S2A Fig), increased over time and peaked at 5 dpi, in an expanding bacterial-dependent manner, usually in close vicinity of the infection foci, particularly after R infection (Fig 1E).

Both *Mabs* R and S were found close to or within eGFP-positive cells near to the injection site at 1 dpi (S2B Fig). At later time points, in both S- and R-infected embryos, a strong eGFP signal was detected in *Mabs*-containing granulomas (Fig 1F and 1G) but not around or within R-abscesses (Fig 1G), the latter consisting essentially of highly replicating extracellular mycobacteria associated with tissue damage [17]. eGFP-positive cells were also surrounding *Mabs* serpentine cords (Fig 1H). Because TNF-α can be produced by numerous cells [23], we attempted to identify the TNF-producing cells in response to *Mabs*, with the double transgenic *Tg*(*tnfα*:*eGFP-F/mpeg1*:*mCherry-F*) or *Tg*(*tnfα*:*eGFP-F/LysC*:*DsRed*) embryos. It was possible to visualize green *tnf-α* expression concomitantly with red macrophages or red neutrophils, respectively. For both R and S infections, cells producing TNF-α co-localized with *Mabs*-containing macrophages, either isolated or within granulomas (S2B Fig and Fig 1I). In sharp contrast, the eGFP signal failed to co-localize with neutrophils (Fig 1J and S2C Fig). The total lack of TNF-positive cells in macrophage-depleted *Tg*(*tnfα*:*eGFP-F*) larvae (S2D Fig), generated after lipo-clodronate injection [17], confirmed macrophages as the primary source of TNF in response to *Mabs* infection.

Taken collectively, these results indicate that TNF is principally produced by macrophages following infection with both *Mabs* variants, from very early phagocytosis after infection to later time points when the characteristic granulomas have appeared.

### Impaired TNF-mediated immunity correlates with severe and lethal infections

**Fig 2:**
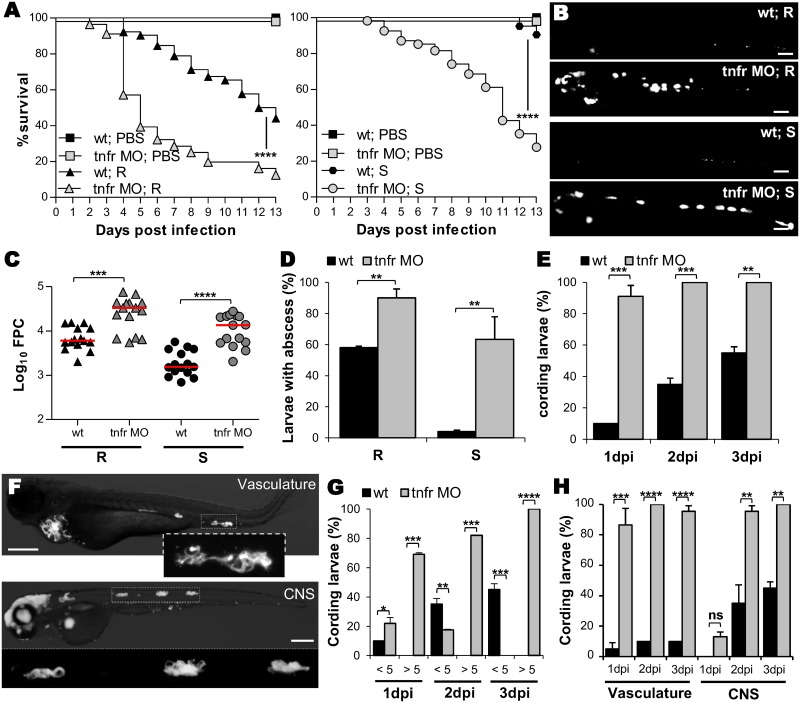
*tnfr* morphants are highly susceptible to *Mabs* infections. (A-H) WT or *tnfr* morphants were *iv* infected with either R (A-H) or S (A-D) variants of *Mabs* (tdTomato, ≈150 CFU). (A) Survival of infected embryos *versus* PBS-injected embryos (n = 90, average of three independent experiments). (B-C) Representative fluorescence images of: (B) Bacterial loads (FPC, two independent experiments, horizontal lines indicate the median values); (C) 3 dpi embryos infected by either R or S variants. Scale bars, 200 μm. (D) Proportion of larvae with abscesses after 13 dpi expressed as mean ± SEM from two independent experiments (n = 40–50). (E) Kinetic of R-cord formation in whole infected embryos. Mean ± SEM from three independent experiments (n = 30). (F) Fluorescence microscopy of *tnfr* morphants exhibiting widespread R-cording in the vasculature and in the CNS at 3 dpi. Scale bar, 200μm. (G-H) Proportion of embryos containing <5 or >5 cords (G) and localization of cords (H) in (E). Error bars represent the SEM. Statistical significance was determined by log-rank test (A), one-tailed Mann-Whitney’s t test (C) or Fisher’s exact test of a contingency table (D-E and G-H).

TNF-α is a multifunctional cytokine playing a pivotal role in the regulation of inflammation and infection *via* the stimulation and engagement of the specific cell surface receptor 1 (TNFR1, ZDB-GENE-040426-2252). To address the role of TNF signaling in *Mabs* infections, loss-of-function experiments for TNFR1 were carried out using a specific morpholino, leading to complete abrogation of the native *tnfr1* mRNA (S3A Fig) and thereby subsequent TNF production (S3B–S3D Fig). Although showing morphological defects (S3A Fig), uninfected *tnfr1* (*tnfr*) morphants exhibited survival rates similar to those of wild type (WT) embryos (Fig 2A). Importantly, TNFR1 impairment led to an increase in the severity of the infection and hyper-susceptibility to R and S variants (Fig 2A). This correlated with higher bacterial burdens as demonstrated by whole embryo imaging (Fig 2B) and fluorescent pixel counts (FPC) (Fig 2C). Imaging of the R- and S-infected *tnfr* morphants showed that the increased bacteremia coincided with the presence of highly-replicating extracellular bacteria, resulting in the rapid development of abscesses (Fig 2B and 2D). Whereas abscesses remain the exclusive attribute of R infections in WT embryos, 60% of S-infected *tnfr* morphants developed abscesses (Fig 2D). Similarly, after R-infection, rapid and massive cord formation occurred in nearly all *tnfr* morphants within 1 dpi (Fig 2E and 2F). At 1 dpi, WT embryos had fewer cords (<5) while 70% of the morphants had high cord numbers (>5) (Fig 2G). Whereas cords developed essentially within the CNS in WT fish, all *tnfr* morphants exhibited widespread cording in the vasculature, in addition to the CNS (Fig 2F and 2H). Thus, hyper-cording of *Mabs* R occurs very rapidly in the absence of TNF-mediated immunity, leading to early larval death, as has been described for *Mycobacterium marinum* in embryos lacking TNF signaling [24].

These results demonstrate the crucial and protective role of the TNFR1-dependent pathway in response to *Mabs* S and R by restricting extracellular multiplication and pathogenesis.

### TNFR1 knockdown inhibits the macrophage bactericidal activity and promotes macrophage death

**Fig 3:**
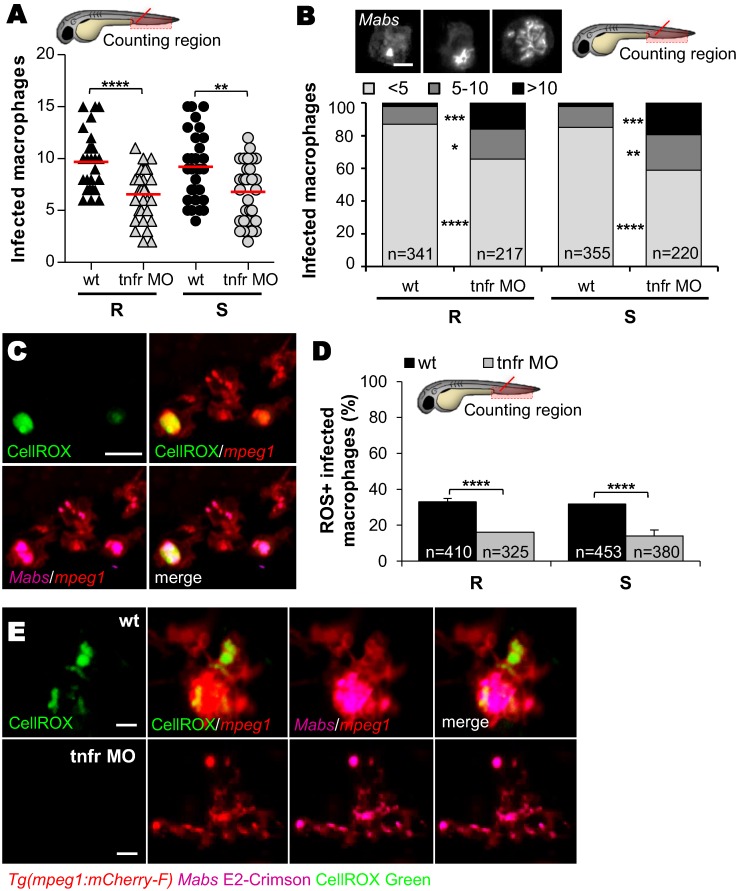
Inhibition of intramacrophage *Mabs* growth correlates with TNF-mediated ROS production. (A-E) WT or *tnfr* morphant *Tg(mpeg1*:*mCherry-F)* embryos were *iv* infected with ≈150 R or S *Mabs* expressing tdTomato (A and B) or E2 crimson (C-E). (A) Number of infected macrophages in the caudal hematopoietic tissue (CHT) at 4 hpi. Horizontal lines indicate the mean values. (B) Average proportions of macrophages containing <5, 5 to 10, or >10 bacteria in the CHT at 1 dpi (n = 20 embryos for each group). Top panel shows confocal images of each representative class of infected macrophages. Scale bar, 5 μm. (C-E) CellRox green (green) staining of ROS production in infected embryos. (C) Confocal microscopy of ROS-positive infected macrophages. Scale bars, 10 μm. (D) Average proportions ROS-producing macrophages within the CHT in 1 dpi embryos (n = 30 embryos for each group). Error bars represent the SEM. (E) Representative fluorescence microscopy of 3 dpi-granuloma. Scale bars, 10 μm. Statistical significance was determined by one-tailed unpaired Student’s t test (A) or Fisher’s exact test of a contingency table (B and D). Data are representative of two independent experiments.

To define how TNF orchestrates the events leading to the *Mabs* pathophysiology, we addressed whether the increased mortality and unrestricted mycobacterial growth in *tnfr* morphants are linked to a defect in macrophage recruitment, phagocytosis and/or bactericidal activity. The ability of macrophages to traffic across the epithelial and endothelial barriers was evaluated following S and R injection into the muscle (S4A Fig) and the otic cavity (S4B and S4C Fig) of *Tg(mpeg1*:*mCherry-F*) larvae. Irrespective of the infection site, the number of early recruited macrophages is comparable in both *tnfr* morphants and WT embryos at 2 hpi, implying that TNF signaling is not required for the early trafficking of macrophages, consistent with previous studies with *M*. *marinum* [24]. Although bacteria were rapidly engulfed by macrophages after infection of *tnfr* morphants (S4A Fig), the number of infected macrophages harboring either R or S bacteria was lower in morphants than in WT embryos at 4 hpi (Fig 3A). Knocking-down TNFR1 expression altered TNF expression (S3B–S3D Fig) and a defective TNF-positive feedback loop seems to be required for the efficient recruitment of macrophages at later stages (S4B Fig) and subsequent phagocytosis. The ability of *tnfr* morphants to develop abscesses in the CNS at later time points after intravenous infection suggests that macrophages remain efficient in transporting and disseminating the bacteria from the bloodstream to deeper tissues (Fig 2).

To assess the contribution of TNF signaling in modulating the mycobactericidal activity of macrophages, the number of intracellular bacteria in individual infected macrophages was determined. The proportion of slightly infected (<5 bacteria), moderately infected (5–10 bacteria) or heavily infected (>10 bacteria) macrophages was enumerated at 1 dpi (Fig 3B). Compared to the WT embryos, the *tnfr* morphants displayed a greater percentage of macrophages in the high burden category. This was true for both R and S variants and suggests that TNF restricts intracellular growth by stimulating the macrophage bactericidal activity. Further confirmation was obtained following staining of the infected embryos with a probe that detects reactive oxygen species (ROS) (CellROX) (Fig 3C). At 1 dpi, ROS-labeled infected macrophages were found in WT embryos (Fig 3D), in agreement with previous reports showing that macrophages can produce ROS to control *Mabs* infections [25,26]. No differences in the proportion of ROS-positive macrophages containing either *Mabs* S or R were noticed (Fig 3D). However, fewer ROS-positive infected macrophages were found in morphants as compared to WT embryos (Fig 3D), and ROS-positive macrophages in granulomas were only seen in WT animals (Fig 3E). Aggregating cells positive for CellROX staining were occasionally observed, but only in WT larvae (S5A and S5B Fig). However, the relatively low numbers of ROS-labeled infected macrophages, reflecting a reduced bactericidal activity in *tnfr* morphants, is unlikely to explain the extreme susceptibility of the *tnfr* morphants to *Mabs* and the very high extracellular bacterial loads. Because macrophage death has been previously shown to release extracellular *Mabs*, we examined the extent of macrophage death in infected larvae [17]. Imaging of the acridine orange (AO)-infected larvae (S6A Fig) and quantitative determination of AO-positive macrophages (S6B Fig) showed the significantly higher numbers of dead infected-macrophages in the *tnfr* morphants at 2 dpi as compared to the WT embryos. As expected, the basal levels of dead macrophages were very low in PBS-injected WT embryos or uninfected *tnfr* morphants (S6A Fig).

Overall, these results indicate that TNF signaling is pivotal in establishing the initial innate immune response by: i) triggering the early bactericidal activity in macrophages and granulomas to restrict intracellular growth; ii) reducing uncontrolled extracellular bacterial growth by preventing macrophage death; and iii) promoting the formation of inflammatory cell aggregates which, in turn, may contribute to amplifying the local inflammation and recruitment of other immune cells [27].

### IL8-dependent neutrophil mobilization during *M*. *abscessus* infection

**Fig 4:**
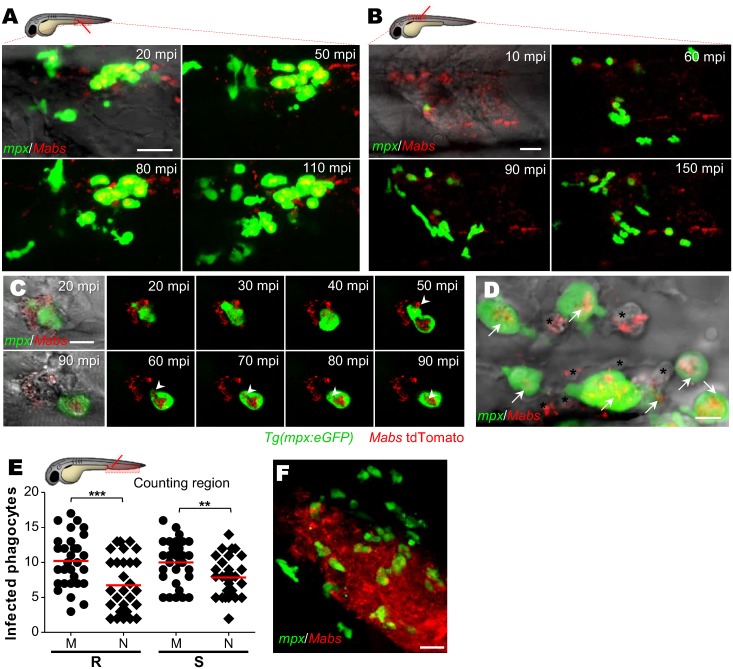
*M*. *abscessus* infections are associated with a massive neutrophil mobilization. (A-D) Confocal live imaging of *Tg(mpx*:*eGFP)* larvae after *iv* (A and D) or intramuscular (B and C) infection with ≈100 *Mabs* R (tdTomato). (A-B) Time-lapse of neutrophils recruitment in caudal vein (A), monitored from 20 mpi to 110 mpi, or into muscle (B) monitored from 10 mpi to 150 mpi. Scale bars, 20 μm. (C) Recruited neutrophils phagocytizing individual mycobacteria. Scale bar, 5 μm. (D) Neutrophils (arrows) and presumptive macrophages (*), containing mycobacteria. Scale bar, 20 μm. (E) Number of infected neutrophils and macrophages counted in CHT at 4 hrs post intravenous infection with both *Mabs* variants (≈150 CFU, three independent experiments, horizontal lines indicate mean values). M, macrophage; N, neutrophils. Statistical significance was determined by one-tailed unpaired Student’s t test. (F) Confocal imaging of neutrophil-bacteria interactions in the context of R-abscess. Scale bars, 20 μm. See also S1 Movie.

Neutrophils are the first line of defense against pathogenic microorganisms and are rapidly recruited to infection sites where they engulf microorganisms and excrete their granule contents [28]. *Mabs*-containing neutrophils were previously identified in granulomas [17] but how these cells are recruited and contribute the immunity against *Mabs* remains unknown. Time-lapse microscopy of neutrophil mobilization in the caudal vein (Fig 4A) or in the muscle (Fig 4B and S1 Movie) of *Tg(mpx*:*eGFP)* embryos revealed a massive influx of neutrophils at the infection site starting at 10–20 min post-infection (mpi). Isolated *Mabs* were rapidly engulfed by neutrophils (Fig 4C and 4D), however the number of neutrophils harboring either R or S bacteria remained lower than the number of infected macrophages at 4 hpi (Fig 4E). Time-lapse microscopy showed a massive mobilization of neutrophils within the deeper CNS infection foci (S7A Fig), especially around abscesses (Fig 4F). Interestingly, the R-abscesses continued to expand, concomitant with a time-dependent disappearance of the neutrophils, reminiscent of the neutropenia (S7A Fig), and high bacteremia occurs prior to larval death as reported in embryos infected with *Shigella* [29].

**Fig 5:**
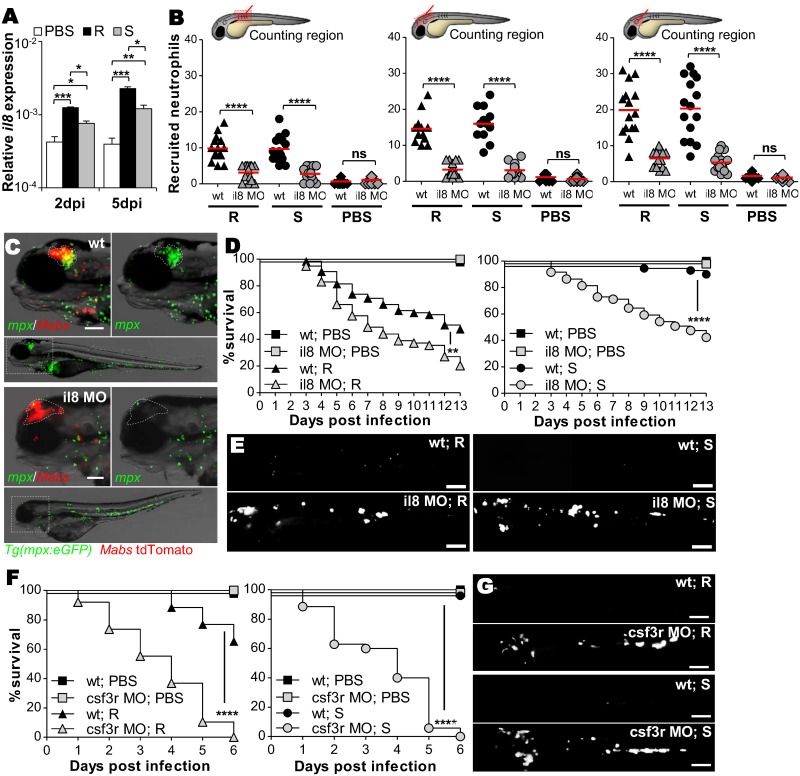
IL8-dependent recruited neutrophils phagocytize *Mabs* at initial sites of infection. (A) qRT-PCR measurement of *il8* transcripts during *Mabs* R- or S-infections (≈150 CFU) in whole embryos at 2 and 5dpi. Mean log_10_ ± SEM of three independent experiments. (B) WT or *il8* morphants *Tg(mpx*:*eGFP)* embryos were infected into the muscle (left), the hindbrain (middle) or the otic vesicle (right) with ≈100 R or S *Mabs*. Number of recruited neutrophils in injection sites at 3 hpi (two independent experiments, horizontal lines indicate the median values). (C) Microscopy showing representative neutrophil recruitment to R-abscesses in the brains of WT *versus il8* morphants *Tg(mpx*:*eGFP)* larvae at 5 dpi. Scale bar, 100 μm. (D and E) WT or *il8* morphants were *iv* infected with either R or S (tdTomato, ≈150 CFU). (D) Survival of R-(left) or S-infected (right) embryos (n = 50–60, average of two independent experiments). (E) Representative images of infected larvae at 3 dpi. Scale bars, 200 μm. (F and G) WT or *csf3r* morphants were *iv* infected with either R or S expressing tdTomato (≈150 CFU). (G) Survival of R-(left) or S-infected (right) embryos (n = 50–60, two independent experiments). (H) Representative images of infected larvae at 3 dpi. Scale bars, 200 μm. Statistical significance was determined by Kruskal-Wallis test with Dunns post-test (A), one-tailed unpaired Student’s t test (B) or by log-rank test (D and F).

IL8 is a central chemokine in neutrophil mobilization from hematopoietic tissues to infection sites. qRT-PCR revealed up-regulation of *il8* early after infection, coinciding with granuloma formation and peaking at 5dpi, with higher levels for R infection than for S infection (Fig 5A). Injection of a morpholino targeting *il8* expression [30] in WT embryos strongly inhibited early neutrophil mobilization into infected muscle, hindbrain or otic cavity (Fig 5B) while leaving the baseline number of neutrophils unchanged, as reported earlier [30]. At later stages of infection, no neutrophils were associated to the CNS abscesses of *il8* morphants, a phenomenon unrelated to neutropenia (Fig 5C). In sharp contrast, while infection of *il8* morphants resulted in strongly impaired neutrophil recruitment in the muscle, the hindbrain and the otic cavity (Fig 5B), mobilization (S8A Fig) and phagocytosis (S8B Fig) of macrophages were unaffected by the *il8* morpholino injection. Importantly, IL8 ablation correlated with reduced larval survival (Fig 5D) and with increased S and R loads (Fig 5E).

The lack of neutrophil recruitment seen with IL8 ablation paralleled the pronounced increase in the bacterial loads, as evidenced by the numerous large abscesses (Fig 5E), suggesting that the absence of neutrophils at the site of infection may be deleterious for the host. To test this hypothesis, specific neutrophil depletion was carried out though injection of *csf3r* morpholino [31], which at the concentration used did not affect the macrophage recruitment (S9A Fig) or macrophage phagocytic activity (S9B Fig). As seen in the *il8* morphants, the *csf3r* morphants showed hyper-susceptibility to both R and S infections with 100% larval mortality at 6 dpi (Fig 5F) and massive extracellular bacteremia (Fig 5G). Overall, these findings indicate that IL8 mediates neutrophil mobilization to the infection sites and plays a critical role in host defense against *Mabs*.

### TNF signaling is crucial to neutrophil recruitment at *M*. *abscessus* infection sites

**Fig 6:**
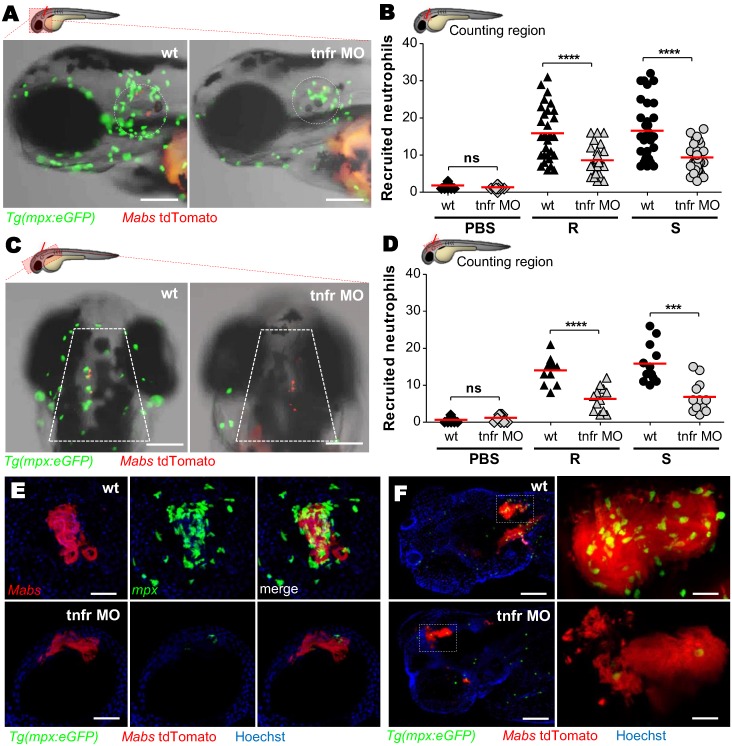
TNF signaling is required for neutrophil recruitment. (A-D) WT or *tnfr* morphant *Tg(mpx*:*eGFP)* larvae were injected in the otic cavity (A-B) or hindbrain (C-D), with both variants of *Mabs* (tdTomato, ≈100 CFU, two or three experiments) and imaged at 3 hpi. Representative images (A and C) and number (B and D) (horizontal lines indicate the mean values) of neutrophils at the infection site. Scale bars, 100 μm. (E and F) Confocal image of neutrophils surrounding a 3 dpi R-cord (E) (Scale bars, 50 μm) or a 5 dpi R-abscess (F) (Scale bars, 100 μm) in *iv* infected WT or *tnfr* morphant *Tg(mpx*:*eGFP)* embryos. Statistical significance was determined by one-tailed Student’s t test (B and D). See also S2–S5 Movies.

TNF orchestrates the early regulation of chemokine induction, including IL8, essential for neutrophil activation and recruitment to inflamed tissues [32]. To address the role of TNF in neutrophil mobilization, local infections were done in the otic (Fig 6A and 6B) and hindbrain (Fig 6C and 6D) cavities of *Tg(mpx*:*eGFP) tnfr* morphants. While neutrophils were rapidly recruited to the infected ear or hindbrain in WT animals, their recruitment was severely reduced in the *tnfr* morphant for both S and R variants (Fig 6A–6D), supporting a major role of TNF in early neutrophil mobilization. Whilst confocal microscopy of the cord/abscess-containing environments demonstrated massive mobilization of neutrophils around cords (Fig 6E and S2 Movie) and abscesses (Fig 6F and S4 Movie) in WT embryos, this was not true in *tnfr* morphants (Fig 6E and S3 Movie, Fig 6F and S5 Movie). The reduced number of neutrophils in *tnfr* morphants (S10A and S10B Fig), was consistent with a study reporting the influence of TNF on hematopoietic stem cell formation [33]. To inquire whether the decreased neutrophil recruitment in *tnfr* morphants is linked to a possible alteration in a basal neutrophil number, we performed a neutrophil mobilization assay using fMLP, a synthetic neutrophil chemoattractant [34]. After injection of fMLP into the otic cavity of *tnfr* morphants, neutrophils were recruited to the injection site to the same extent as in the WT embryos (S10C Fig). Comparable results were also obtained following injection of recombinant IL8 into the *tnfr* morphants (S10D Fig). Thus, the decreased neutrophil recruitment in *tnfr* morphants (Fig 6) is due neither to the general reduction of the neutrophilic population nor to nonspecific effects of the *tnfr* morpholino used.

Together, these results further position TNF as a critical mediator in initiating the early and late phases of neutrophil recruitment and substantiate the crucial role of neutrophils in controlling *Mabs* infections.

### Defective TNF signaling abrogates granuloma formation to *M*. *abscessus*

**Fig 7:**
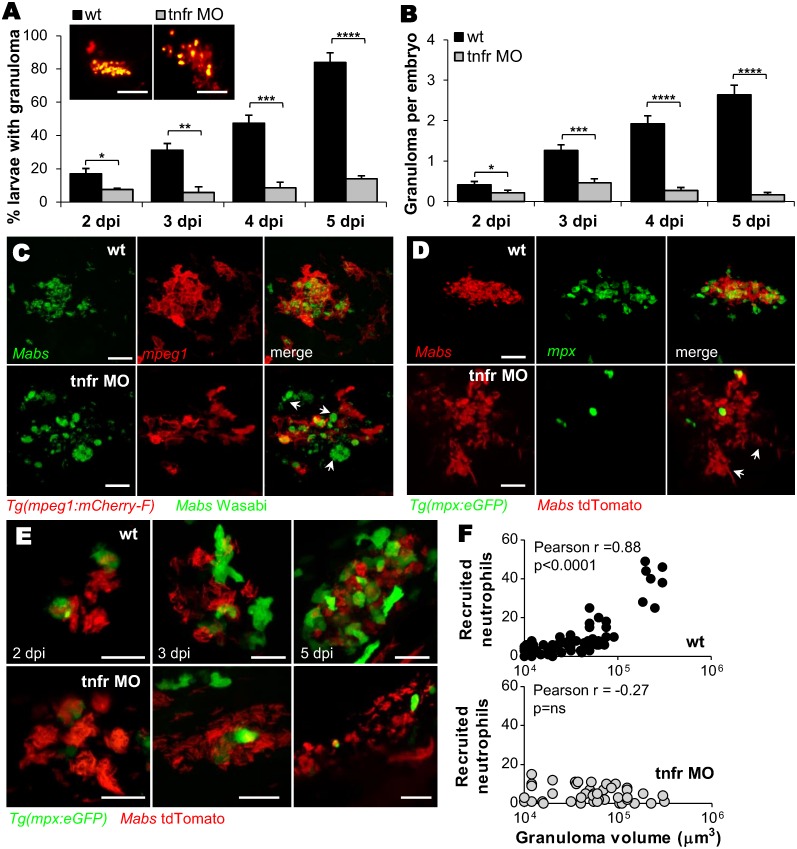
Recruitment of neutrophils is crucial for granuloma development. (A and B) WT or *tnfr* morphants were *iv* infected with R *Mabs* (tdTomato, ≈150 CFU). Kinetics of granuloma formation (A) and mean ± SEM number of granuloma per infected embryos (B) in (A); (n = 30–45, three independent experiments). The top panel shows confocal images of representative granuloma. Scale bars, 100 μm. (C) Confocal images showing a representative 3 dpi granulomas in WT *versus tnfr1* morphant *Tg(mpeg*:*mcherry-F)* embryos *iv* infected with S expressing Wasabi. Arrows indicate extracellular aggregates. Scale bars, 15 μm. (D) Confocal microscopy showing representative granulomas in WT and *tnfr1* morphant *Tg(mpx*:*eGFP)* embryos *iv* infected with R expressing tdTomato at 4 dpi. Arrows indicate extracellular cords. Scale bars, 30 μm. (E-F) Confocal microscopy monitored WT or *tnfr* morphant *Tg(mpx*:*eGFP)* embryos that were *iv* infected with R (tdTomato, ≈150 CFU). (E) Representative kinetics of neutrophil mobilization during granuloma formation. Scale bars, 20 μm. (F) Number of neutrophils recruited to WT (up) or TNFR1-depleted (down) granuloma as a function of granuloma volume. Statistical significance was determined by Fisher’s exact test of a contingency table (A), one-tailed unpaired Student’s t test (B) or Pearson correlation (F). See also S6 and S7 Movies.

Despite the co-existence of macrophages and neutrophils in *Mabs*-induced granulomas [17], the importance of neutrophils in the maintenance and/or integrity of this organized cellular structure remains elusive. Monitoring the kinetics of granuloma development revealed that granulomas induced by both R- and S-variants appeared at 2 dpi and expanded in most WT embryos at 5 dpi (Fig 7A and 7B and [17]). In sharp contrast, the granuloma-like structures in the *tnfr* morphants appeared as poorly delimited loose cellular aggregates (Fig 7A–7E and S11 Fig), similar to those previously documented in TNF defective *M*. *marinum* [24], and highlighting the absolute requirement of a functional TNF pathway for granuloma formation. Confocal microscopy revealed a more open and disjointed structure with the presence of high numbers of extracellular bacterial aggregates (Fig 7C), prompting us to examine whether the increased proportion of dead macrophages in infected *tnfr* morphants (S6A and S6B Fig) may contribute to the morphologically altered granulomas and the reduced granuloma numbers. As shown in S6C Fig, despite the higher proportion of dead macrophages in the *tnfr* morphants as compared to the WT embryos, there was no correlation with the number of “defective” granulomas found in the *tnfr* morphants. While granulomas in WT embryos contained numerous neutrophils, they were nearly absent in the corresponding structures in *Tg(mpx*:*eGFP) tnfr* morphants, which were characterized by substantial numbers of extracellular bacteria/cords (Fig 7D, S6 and S7 Movies). Time-lapse monitoring (Fig 7E) and determination of the number of infected neutrophils recruited to the granulomas (Fig 7F) established a linear relationship between the number of recruited neutrophils and the granuloma volume in WT embryos. In contrast, *tnfr* morphants exhibited an important lack of neutrophils (Fig 7E) and no linear correlation with the size of the granuloma-like structures could be established (Fig 7F), suggesting a direct participation of neutrophils in elaborating and shaping *Mabs* granulomas.

### IL8/TNF-dependent neutrophil mobilization is required for granuloma formation

**Fig 8:**
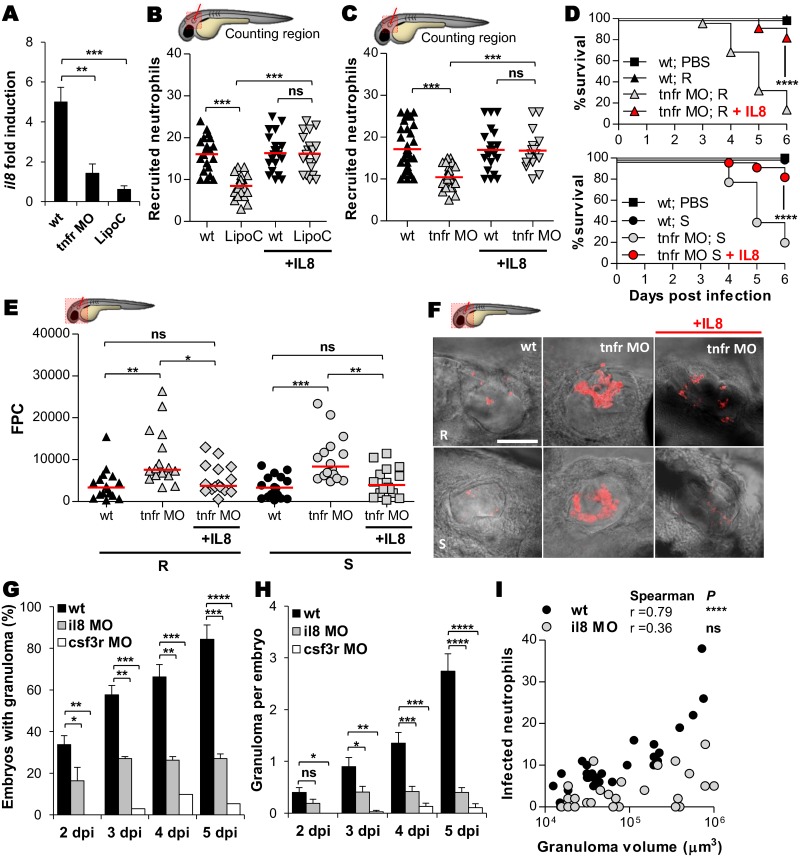
IL8-dependent mobilization of neutrophils is crucial for controlling *M*. *abscessus* infection and elaborating granulomas. (A) qRT-PCR of *il8* (normalized to *ef1α*) upon *Mabs* infection in R *Mabs iv* infected WT, *tnfr* morphants or LipoC embryos (≈150 CFU). Fold induction to PBS- injected animals at 3 dpi. Data are mean ± SEM of two independent experiments. (B and C) WT, LipoC embryos (B) or *tnfr* morphants (C) *Tg(mpx*:*eGFP)* embryos were infected into the otic vesicle with ≈150 R *Mabs* and treated with IL8 injection. Mean number of recruited neutrophils into the otic cavity in response to *Mabs*/IL8 injection at 3 hpi. Each symbol represents individual embryos and horizontal lines indicate the mean values. (D-F) WT or *tnfr* morphants were injected into the otic cavity with either ≈150 R or S variants expressing tdTomato and treated with IL8 injection. (D) Survival of R-(up) or S-infected (down) embryos (n = 50–60, two independent experiments). (E-F) Bacterial loads (FPC, two independent experiments, horizontal lines indicate the mean values) (E) and representative confocal images (F) of 3 dpi embryos. Scale bars, 200 μm. (F and G) WT, *il8* or *csf3r* morphants were *iv* infected with R *Mabs* (tdTomato, ≈150 CFU). Kinetics of granuloma formation (G) and number of granuloma per infected embryos (H) assessed at 3 dpi. Mean ± SEM from two independent experiments (n = 40). (I) Number of neutrophils recruited to WT or IL8-depleted granulomas as a function of the granuloma volume at 2 dpi. Statistical significance was determined by Kruskal-Wallis test with Dunns post-test (A and H), ANOVA with Tukey post-test (B, C and E), log-rank test (D), Fisher’s exact test of a contingency table (G) or Spearman correlation (I).

That TNF modulates the early mobilization of neutrophils into granulomas led us to determine whether TNF depletion influences IL8 expression. Quantitative RT-PCR revealed that, while *Mabs* stimulated *il8* expression levels in WT larvae, its expression was severely decreased in the TNFR1-depleted larvae (Fig 8A) and was even further reduced in macrophage-depleted larvae following injection of lipoclodronate (Fig 8A), indicating that macrophages are part of the pathway that triggers IL8 release in response to *Mabs* infections. This suggests that in WT embryos, TNF production by macrophages (Fig 1I) governs IL8-driven chemotaxis, and thus the absence of TNF signaling profoundly restricts neutrophil mobility. Having confirmed that macrophages are required for IL8 production, we next examined whether neutrophil mobilization to the infection site is dependent on macrophages. Neutrophil recruitment was dramatically reduced in the macrophage-depleted larvae (Fig 8B), demonstrating that macrophages are key players in neutrophil mobilization in response to *Mabs* infection. In addition, while neutrophil recruitment is strongly impaired in the absence of either macrophage or TNFR signaling, the injection of exogenous IL8 fully rescued neutrophil mobility (Fig 8B and 8C) and restored survival of *tnfr* morphants infected with R or S in the otic cavity (Fig 8D). This correlated with decreased bacterial loads compared to untreated *tnfr* morphants, as evidence by the determination of the FPC (Fig 8E) and fluorescence microscopy (Fig 8F). These findings highlight the immune-protective role of IL8-dependent neutrophil mobilization during *Mabs* infections. Furthermore, granuloma formation was severely impaired in *il8* morphants and, consistent with these findings, granuloma formation was abrogated in the neutrophil-depleted *csf3r* morphants (Fig 8G and 8H). Similarly to *tnfr* morphants (Fig 7F), *il8* morphants exhibited neutrophil-poor granulomas (Fig 8I), further supporting the direct participation of neutrophils in elaborating and shaping *Mabs* granulomas.

Overall, these results emphasize the absolute requirement of an IL8/TNF-dependent neutrophil mobilization for granuloma formation and control of *Mabs* infections.

## Discussion

**Fig 9:**
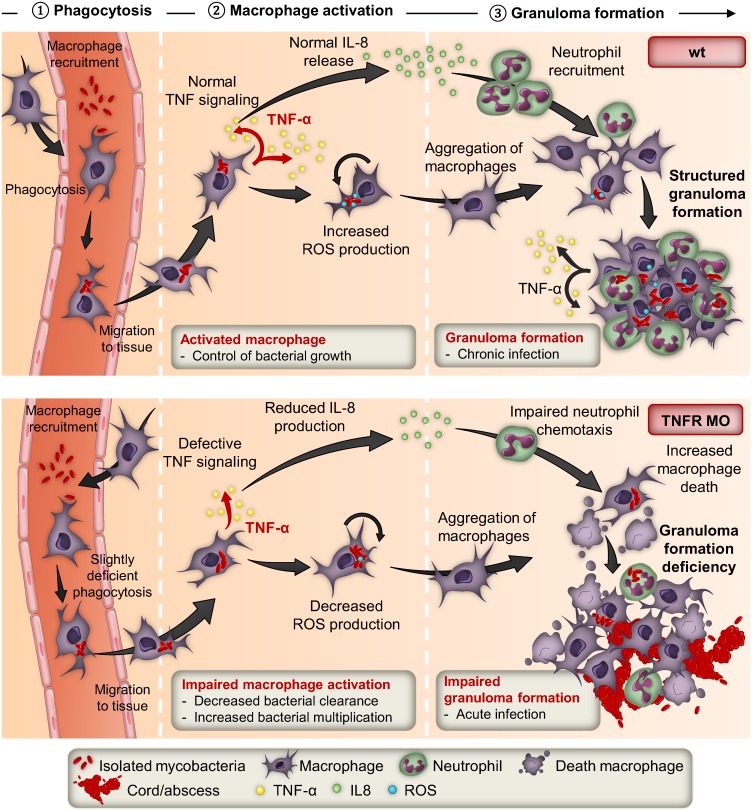
IL8- and TNF-dependent neutrophil mobilization in *Mabs* granuloma formation and immunoprotection. The upper panel summarizes the contribution of the TNF/IL8 axis in the early and late response to *Mabs* infection leading to a controlled and chronic infection. The lower panel illustrates the detrimental effects of impaired TNF signaling that, through disruption of the IL8 production and associated defect in neutrophil mobilization, leads to impaired granuloma formation, acute infections and larval death.

Herein, we report the first stepwise dissection study of the immune control of *Mabs* using a non-invasive visualization approach with special emphasis on the inflammatory response. The spatiotemporal immunopathological events (Fig 9) can be summarized as follows: i) rapid engulfment of *Mabs* by macrophages; ii) TNF release by activated macrophages, leading to ROS production and intracellular killing of *Mabs*, and IL8-driven chemotaxis that guides neutrophils to the infection site; iii) proficient granulomatogenesis and development of chronic infections. In contrast, defective TNF signaling correlates with i) impaired macrophage activation with increasing intramacrophage bacterial loads and disruption of IL8 production, resulting in impaired neutrophil recruitment; ii) absence of *bona fide* granulomas. Additionally, the increased macrophage death releases free bacilli that multiply extracellularly in an uncontrolled manner, resulting in mycobacterial cords that prevent phagocytosis by macrophages and neutrophils [17], acute infections and larval killing.

*Mab*s infections are characterized by growth in highly inflamed tissues, suggesting a role for neutrophils in the host response. Supporting this hypothesis, patients with CF, a disease that is dominated by persistent neutrophil-mediated inflammation, are particularly susceptible to *Mabs* in addition to other extracellular pathogens [4,35]. However, despite the prevalence of neutrophils in *Mabs* infections, information regarding the neutrophil response is scarce. Previous work suggested that human neutrophils mediate killing of *Mabs*, but phagocytosis was reduced when compared to *Staphylococcus aureus*, another important CF pathogen [36]. In experimental models of *Mabs* infection, the presence of increased numbers of neutrophils is associated with a worse response to *Mabs* [37]. We assessed here the *in vivo* capacity of neutrophils to migrate in response to *Mabs*, to engulf the bacilli and to participate directly to granuloma formation in infected zebrafish. Our results are distinct from those with *Mycobacterium tuberculosis* or *M*. *marinum* where neutrophils appear to be less mobilized [34,38]. *Ex vivo* infections of human lung tissues indicated that neutrophils had a greater tendency to phagocytize *Mabs* than *M*. *tuberculosis* and that *Mabs* has a higher capacity to induce the migration of neutrophils than other mycobacterial species [38]. Additionally, while neutrophils are unable to kill virulent strains of *M*. *tuberculosis* or *M*. *marinum* [39,40], they appear to be important for controlling virulent and avirulent *Mabs* because neutrophil-depleted embryos are extremely susceptible to *Mabs* infection. The mobilization of neutrophils toward the large cords and abscesses is also in agreement with recent studies reporting that neutrophils, through a microbial size-sensing mechanism, tailor their antimicrobial responses to pathogens based on microbial size [41].

Recent studies have indicated that *M*. *tuberculosis*-infected neutrophils can be considered as biomarkers for poorly controlled mycobacterial replication and are associated with severity in human tuberculosis [42]. Our data show that infected neutrophil-depleted embryos exhibit increased CNS pathology, high mycobacterial loads, decreased survival rates and reduced granuloma numbers. They also support the crucial role of IL8 in the control of *Mabs* at the infection site due to its function as the main mediator of neutrophil mobilization to the infection site, and through this recruitment of neutrophils, the formation of granulomas. Indeed, the absence of granulomas caused by *il8* or *tnfr* knock-down was associated with compromised survival and reduced bacterial clearance of *Mabs*, phenotypes that were restored upon injection of recombinant IL8. Despite the important role of macrophages in IL8 signaling, production of IL8 was not completely abrogated in the macrophage-depleted embryos, suggesting that other cell types may also contribute to IL8 production.

Our results thus shed new light on the role of neutrophils in early granuloma formation, integrity and maintenance, and reveals striking differences with the dynamics of granuloma formation in the *M*. *marinum* zebrafish model, where granulomas contribute to early bacterial growth and expansion of the infection [43]. Although one cannot exclude the possibility that *Mabs* exploits the granuloma to manipulate host immune responses for its own benefit as suggested for *M*. *marinum*, our conclusions support the primary role of granulomas in preventing a widespread expansion of *Mabs* in the extracellular milieu. Granuloma-defective embryos (*tnfr*, *il8* or *csf3r* morphants) were all hyper-susceptible to S and R infections with pronounced larval mortality and unrestricted extracellular bacterial growth. Both R and S strains simulated granuloma formation at comparable levels [17], indicating that granuloma formation is not correlated with virulence of *Mabs*. In contrast, granuloma formation with *M*. *marinum* is linked to virulence, as demonstrated using the attenuated RD1-deficient mutant [44]. Apparently, the distinctive kinetics and functions of granulomas are species-specific and conclusions drawn from one mycobacterial species cannot be extrapolated to others.

Furthermore, while *M*. *marinum*-infected embryos deficient in TNF signaling showed increased granuloma formation in early stages of infection [24], after *Mabs* infection of *tnfr* morphants the granuloma formation was almost completely abrogated. Instead of compact organized granuloma, *Mabs* infection of *tnfr* morphants elicited only the formation of disorganized cellular structures that consisted of aggregated macrophages. A similar result was seen in TNF-α KO mice where impaired *Mabs* control was associated with profound alteration of granuloma formation [16]. Why would TNF exert distinct responses when stimulated with different mycobacterial species? It is well known that the mycobacterial cell envelope possesses a large panel of surface-exposed glycolipids, some of which are particularly granulomatogenic. Although the overall architecture of the cell wall is conserved among mycobacteria, each species can be typified by a specific lipid/glycolipid signature, and subtle variations in the lipid composition, structure and size can affect granulomatogenesis. Other effectors, such as the ESX-1 are also required for efficient granuloma formation with *M*. *tuberculosis* and *M*. *marinum* [44,45]. While the loss of RD1 in a *M*. *marinum* is associated with altered early aggregation of infected macrophages and a delay in granuloma formation [44], *Mabs*, which naturally lacks RD1, induces “normal” granuloma formation in embryos [17], adult zebrafish [46] and in mice [16]. *M*. *marinum* mutants with reduced granuloma formation were also found to be defective in EspL, located in the ESX-1 cluster [47]. *Mabs* presents two ESX-like loci [48] but whether these clusters participate in *Mabs*-induced granulomas and pathogenesis awaits further investigation. The identification of the *Mabs*-specific effectors participating in granuloma formation would contribute to our knowledge of *Mabs* pathogenicity and could foster the development of needed therapeutic interventions, as *Mabs* is refractory to most antimicrobials.

In conclusion, we report here new and unexpected insights into several aspects of *Mabs* immunopathogenesis by demonstrating the crucial role of TNF signaling in a continuum of effects starting from limiting intracellular bacterial multiplication to the induction of granulomas that together exert a protective effect by limiting *Mabs* dissemination. Through its orchestration of the inflammatory cytokine/chemokine network, dominated by IL8 production, TNF modulates the engagement of neutrophils to the infection site and their subsequent recruitment to granulomas, which are essential for control of the early and later stages of *Mabs* infection, respectively. This explains why immunosuppressive TNF therapy increases the risk of *Mabs* infections [49]. Airways of CF patients are characterized by a severe inflammation [50] but whether this directly impacts on subsequent *Mabs* colonization is difficult to predict. The airways of these patients are chronically colonized with complex, polymicrobial infections [51] and these polymicrobial communities promote intricate inter-microbial and host-pathogen interactions which alter the lung environment, impact the response to treatment, and drive the course of the disease. In addition, the relationship between abnormal CFTR expression and the predisposition of CF patients to chronic *Mabs* infections remains elusive, but the results presented here suggest that it would be interesting to determine whether the CFTR defect has a detrimental effect on neutrophil-mediated immunity.

## Materials and Methods

### Zebrafish lines and ethics statement

Zebrafish experiments were conducted in accordance with the guidelines from the European Union for handling of laboratory animals (http://ec.europa.eu/environment/chemicals/lab_animals/home_en.htm) and approved by the Direction Sanitaire et Vétérinaire de l'Hérault et Comité d'Ethique pour l'Expérimentation Animale de la région Languedoc Roussillon under the reference CEEA-LR-1145. For zebrafish anaesthesia procedures, embryos are immersed in a 270 mg/L Tricaine solution in osmotic water. When required, larvae were cryo-anesthetized by incubation on ice for 10 min and then euthanized using an overdose of Tricaine (500 mg/L). Experimental procedures were performed using the *golden* zebrafish mutant [52], along with transgenic lines: *Tg(mpx*:*eGFP)i114* [53] or *Tg*(*LysC*:*DsRed*)*nz5* [54], harboring either green- or red-fluorescent neutrophils, respectively; *Tg*(*mpeg1*:*mCherry-F*)*ump2* [17], harboring red-fluorescent macrophages; and *Tg*(*tnfα*:*eGFP-F*)*ump5* [22], which allows visualization in green of the transcriptomic expression of *tnf-α* under appropriate stimulatory conditions.

### Bacterial strains and systemic or local infections in zebrafish embryos

R and S variants of *M*. *abscessus sensu stricto* strain CIP104536^T^ (ATCC19977T) carrying pTEC15 (Addgene, plasmid 30174), pTEC27 (Addgene, plasmid 30182) or pTEC19 (Addgene, plasmid 30178) that express green fluorescent protein (Wasabi), red fluorescent protein (tdTomato) or bright far-red fluorescent protein (E2-Crimson), respectively, were prepared and microinjected in zebrafish embryos, according to procedures described earlier [17,55]. Briefly, systemic infections were carried by the injection of 100–200 CFU into the caudal vein of 30 hours post-fertilization (hpf) embryos. For phagocyte recruitment assays, 100 CFU were injected locally into the otic vesicle, the muscle or the hindbrain compartments of 3 days post-fertilization (dpf) larvae or into the caudal vein of 2 dpf embryos.

### Neutrophils recruitment assay

Neutrophil recruitment was elicited in zebrafish embryos through injection of the f-Met-Leu-Phe (fMLP, Sigma-Aldrich) or recombinant human IL-8 (rhIL-8, R&D Systems, Inc.), chemoattractants previously described [34]. IL-8 (15–25 pg) or 3 nl of 300 nM fMLP were injected into the otic cavity of 3 dpf larvae. The quantity of recruited neutrophils was determined at the injection site at 3 hpi using fluorescence microscopy.

### Morpholinos and lipo-clodronate injections

Morpholinos purchased from Gene Tools were injected into 1–4 cell stage embryos. *tnfrsf1a* splice-blocking morpholinos targeting TNF receptor 1 (5’-GGAAGCATGAGGAACTTACAGTTCT-3’) were used at a concentration of 0.5 mM. The efficiency of gene knockdown was confirmed by RT-PCR with the following primers for both sides of the morpholino target sequence: *tnfrsf1a*, CCCGCATGCTCCACGTCTCC and TTATAGCGGCCGCCCGACTCTCAAGCTTCA. The zcxc*l8* splice blocking morpholino for IL8 knock-down (5’-TATTTATGCTTACTTGACAATGATC-3’) was prepared and injected as described earlier [30]. To generate neutrophil depleted-embryos, *csf3r* translation morpholino (5’-GAAGCACAAGCGAGACGGATGCCA*T-3’*) targeting the *csf3r* gene was used [31]. For the selective depletion of macrophages into embryos, lipo-clodronate (Lipo-C) [56] was injected into the caudal vein of 24 hpf embryos as previously reported [17,55].

### ROS production and cell death detection

Dead cells in living zebrafish embryos were detected using Acridine Orange (AO), as described [17]. For detecting ROS, living embryos were soaked in 5 μM CellROX Green Reagent (Invitrogen) in Hanks’s Balanced Salt Solution (HBSS) for 30 min at 28.5°C, followed by two washes in HBSS, then transferred into a dish for fluorescent microscopic observation and analyses.

### RNA isolation and qRT-PCR

To determine cytokine/chemokine expression levels, total RNA from a pool of 10–15 larvae per biological experiment was isolated using the Nucleospin RNAII kit (Macherey-Nagel). cDNA synthesis was performed with M-MLV reverse transcriptase (Invitrogen) and then quantitative RT-PCR was performed using a homemade SYBR Green mix on a LightCycler 480 instrument (Roche) as described [57] with the following pairs of primers (sense and antisense): *ef1α*, TTCTGTTACCTGGCAAAGGG and TTCAGTTTGTCCAACACCCA; *tnfα*, TTCACGCTCCATAAGACCCA and CCGTAGGATTCAGAAAAGCG; *il8*, CCTGGCATTTCTGACCATCAT and GATCTCCTGTCCAGTTGTCAT; *ifnγ2*, TGCACACCCCATCTTCCTGCGAA and GTGTTGCTTCTCTATAGACACGCTT; *il1β*, TGGACTTCGCAGCACAAAATG and GTTCACTTCACGCTCTTGGATG. Each experiment was run in triplicate. qRT-PCR datas were calculated using the ΔCt or ΔΔCt method and normalized to the housekeeping gene *ef1α*.

### Microscopy

To quantify bacterial loads, granulomas, cords and neutrophil recruitment, infected larvae were tricaine-anesthetized, positioned on 35-mm dishes, immobilized in 1% low-melting-point agarose and covered with water containing tricaine. Bright-field and fluorescence pictures of live infected embryos were taken with a Zeiss microscope equipped with a Zeiss Plan Neo Fluor Z 1x/0.25 FWD objective and a Axiocam503 monochrome (Zeiss) camera, with acquisition and processing using ZEN 2 (blue edition). Evaluation of intracellular mycobacterial growth, enumeration of macrophages recruitment, phagocytosis, mortality and granuloma organization, infected embryo were prepared for fixed microscopy. Animals were tricaine-anesthetized and fixed overnight at 4°C in 4% (vol/vol) paraformaldehyde in PBS, then washed twice in PBS, and transferred gradually from PBS to 50% (vol/vol) glycerol for microscopic observation. Confocal microscopy was performed using a Leica SPE upright microscope with 40x ACS APO 1.15 oil objective. Images were captured by LAS-AF software (Leica Microsystems).

### Image processing and analysis

Overlays of fluorescent and bright-field images and 2D reconstructions of images stacks were produced, assembled and adjusted using LAS-AF software or GIMP 2.6 freeware. Three-dimensional volume reconstitutions and movies were performed using Imaris 7.0 software (Bitplan AG).

### Statistical analyses

Statistical analyses were performed using Prism 4.0 (Graphpad, Inc) or R 3.0.3 and detailed in each figure legend. *p< 0.05; **p< 0.01; ***p<0.001; ****p< 0.0001; ns, not significant (p≥ 0.05).

## Supporting Information

S1 Fig Inflammation following *Mabs* infections. IL-1β and IFN-γ2 pro-inflammatory cytokines response during *Mabs* infection. PBS, ≈150 *Mabs* R or S variants were *iv* injected. Quantitative RT-PCR using *ef1a* as a reference gene was performed to measure the relative expression of these cytokines in whole embryo assessed at 2 and 5 dpi. Results are presented as mean log_10_ ± SEM of three independent experiments and statistical significance was determined by Kruskal-Wallis test with Dunns post-test.
(TIF)

S2 Fig Early TNF-α Response Induction following *Mabs* infections. (A) *Tg*(*tnf-α*:*eGFP-F*) embryos were infected into caudal vein with ≈100 *Mabs*-expressing tdTomato. Microscopy showing the representative expression of *tnf-α* close to the injection site at 2 hpi. Scale bar, 300 μm. (B-C) Fluorescence microscopy analysis of the GFP expression in *Tg*(*tnf-α*:*eGFP-F/mpeg1*:*mCherryF*) (B) or *Tg*(*tnf-α*:*eGFP-F/LysC*:*DsRed*) (C) double transgenic embryos at 1 day following intravenous infection by either ≈100 R- or S-expressing E2-Crimson. (B) Close-up to the tip of the tail revealing that the transcriptomic *tnf-α* expression is detected in infected macrophages or in macrophages close to the infected tissue. Scale bars, 200 μm. (C) Confocal images of a single *Mabs*-infected macrophage (yellow arrow) close to the injection site induced a strong *tnf-α* expression. Neutrophils are indicated with white arrows. Scale bar, 10 μm. (D) *tnf-α* expression was tested in *Tg(tnf-α*:*GFP-F)* zebrafish embryo in absence of macrophages (lipo-clodronate injection). Embryos were infected with *Mabs* (R variant, ≈100 CFU) in the otic cavity and the proportion of infected embryos with eGFP-positive cells at 2 hpi counted. Graphs represent the mean value of two independent experiments (n = 10).
(TIF)

S3 Fig *Tnfr1* morphants generation. (A-D) Efficiency of morpholino against zebrafish TNFR1. (A) Injection of splice-site blocking antisens morpholinos targeting the TNF receptor 1 (*tnfr*) leads to a total absence of native *tnfr1* transcript. Comparison between WT embryos and *tnfr* morphants transcripts (2 dpf) reveals the complete absence of native transcript. Bright-field microscopy image comparing the whole morphology appearance of WT embryos *versus tnfr* morphants at 2 dpf, showing that morpholino knockdown injection produces a moderate hypomorph phenotype: short body length, big yolk sac, smaller swim bladder, small eyes, reduced pigmentation, hindbrain defects, somites poorly organized and epidermic alterations. Scale bars, 200 μm. (B-D) To check the effect of *tnfr1* loss-of-function on the *tnf-α* production, PBS or tdTomato-*Mabs* (R variant, ≈100 CFU) were injected intravenously (B) or into the otic cavity of either WT or *tnfr* morphants *Tg(tnf-α*:*eGFP-F)* larvae (C-D). (B) qRT-PCR of *tnfα* (normalized to *ef1α*) upon *Mabs* infection. Fold induction compared to entire PBS- injected fishes at 3 dpi. Error bars indicate SEM. (C) Bright-field and fluorescence overlay microscopy showing the real-time visualization of the transcriptional *tnf-α* expression close to the injection site assessed at 2, 4 and 6 hpi. Scale bars, 100 μm. (D) Number of *tnf-α* positive cells per infected larvae evaluated at 2 hpi using confocal microscopy. Each symbol represents individual embryos and horizontal lines indicate the median values. (B and D) Statistical significance was assessed by one-tailed Mann-Whitney’s t test. TNF-α expression subsequent to the infection is impaired in *tnfr* morphants. Results are presented as average number from two experiments.
(TIF)

S4 Fig Ablation of TNF signaling does not affect the early chemoattraction of macrophages to localized *Mabs* infections. To evaluate the effect of absence of TNF signaling on early and late macrophages recruitment, WT or *tnfr* morphants *Tg(mpeg1*:*mCherry-F)* larvae were injected with either PBS or Wasabi-expressing *Mabs* (R variant) into the muscle (A) or otic cavity (B-C), monitored and imaged using confocal microscopy to measure macrophage recruitment. (A) Representative confocal microscopy of macrophages recruitment into the infected muscle at 2 hpi (dotted line outlines 2 somitic muscles). Scale bars, 100 μm. (B-C) Dynamic of macrophage recruitment at the infection site assessed at 2, 4 and 6 hpi (Scale bars, 50 μm) (B) and number of recruited macrophages at 2 hpi (C). Results are presented as average number from two experiments. Each symbol represents individual embryos and horizontal lines indicate mean values. Significance was assessed by one-tailed unpaired Student’s t test. In both WT- and *tnfr* morphant-infected animals, macrophages are recruited towards bacteria at the same rate at early time post-infection. However, while the number of newly recruited macrophages increased progressively in WT larvae from 2 hpi to 6 hpi, the number of recruited macrophages remains constant in *tnfr* morphants.
(TIF)

S5 Fig ROS production by hematopoietic cells following systemic *Mabs* infection. Evaluation of ROS induction in infected embryos using CellROX Green fluorescent. *Tg(mpeg1*:*mCherry-F)* (A) or *Tg(LysC*:*DsRed)* (B) were *iv* infected with E2 Crimson-expressing *Mabs* (R variant) and monitored for the ROS detection. (A) Confocal microscopy of agglomerates of hematopoietic cells display ROS production. Scale bar, 15 μm. Arrows indicate ROS-positive macrophages. (B) Mobilization of neutrophils close to infected tissue with agglomerates of hematopoietic cells producing ROS is revealed by confocal microscopy. Scale bar, 20 μm.
(TIF)

S6 Fig Absence of TNF signaling promotes macrophages death. (A and B) WT or *tnfr* morphants *Tg(mpeg1*:*mCherry-F)* were *iv* infected with either R or S variants of *Mabs* expressing E2 Crimson (≈150 CFU) and stained for dead macrophages with acridine orange (AO). (A) Representative microscopy showing the dead macrophages in 2 dpi R-infected embryos. Scale bars, 80 μm. (B) Number of dead infected macrophages evaluated using confocal microscopy at 2 dpi. Each symbol represents individual embryos and horizontal lines indicate mean values. (C) WT, *il8* or *csf3r* morphants *Tg(mpeg1*:*mCherry-F)* were *iv* infected with either R or S variants (≈150 CFU) and stained for dead macrophages with acridine orange (AO). Statistical significance was determined by one-tailed Student’s t test (B) or Pearson correlation (C). Results are presented as the average number from two experiments.
(TIF)

S7 Fig Behavior of neutrophils during *Mabs* infection. To investigate the behavior of neutrophils following the systemic *Mabs* infection, either R- or S-tdTomato (≈150 CFU) were *iv* injected in *Tg(mpx*:*eGFP)* embryo. Infected embryos were monitored and imaged at different time points following injection to monitor neutrophils recruitment at the infection foci. Bright-field and fluorescence overlay image showing representative recruitment of neutrophils following infections. Scale bars, 200 μm.
(TIF)

S8 Fig Ablation of IL8 signaling does not affect the early activity of macrophages. WT or *il8* morphants *Tg(mpeg1*:*mCherry-F)* larvae were injected with either PBS or R- or S-tdTomato (≈150 CFU) into the otic cavity (A) or the caudal vein (B) and monitored for macrophage recruitment and phagocytosis using confocal microscopy. (A) Number of recruited macrophages into the otic cavity at 2 hpi. (B) Number of infected macrophages in the CHT at 4 hpi. (A-B) Significance was assessed by one-tailed unpaired Student’s. Results are presented as average number from two experiments. Each symbol represents individual embryos and horizontal lines indicate mean values.
(TIF)

S9 Fig Neutrophil depletion does not interfere with early macrophage activity. WT or *csf3r* morphants *Tg(mpeg1*:*mCherry-F)* larvae were injected with either PBS or R- or S-tdTomato (≈150 CFU) into the otic cavity (A) or the caudal vein (B) and monitored for macrophage recruitment and phagocytosis using confocal microscopy. (A) Mean number of recruited macrophages into the otic cavity at 2 hpi. (B) Mean number of infected macrophages in the CHT at 4 hpi. (A-B) Significance was assessed by one-tailed unpaired Student’s. Results are presented as average number from two experiments. Each symbol represents individual embryos and horizontal lines indicate mean values.
(TIF)

S10 Fig Distribution and mobilization of neutrophils in *tnfr1* morphants. (A) Representative bright-field and fluorescence overlay image of WT *versus tnfr* morphants *Tg(mpx*:*eGFP)* embryos at 2 dpf. Scale bars, 200 μm. (B) Quantification of basal number of neutrophils in whole (left) or detailed in the head, in the trunk and tail (right) of 2 dpf embryos (n = 12). Graphs represent the mean ± SEM. (C and D) Mean number of recruited neutrophils into the otic cavity in response to mock, fMLP (C) or IL8 (D) injection in WT and *tnfr* morphants *Tg(mpx*:*eGFP)* embryos monitored at 3 hpi. Each symbol represents an individual embryo and horizontal lines indicate the mean values. Significance was assessed by one-tailed unpaired Student’s t test comparing both embryos per category (B) or by ANOVA with Tukey post-test (C and D). (B-D) Results are representative of two independent experiments.
(TIF)

S11 Fig TNF signaling is crucial for the granuloma development. WT or *tnfr* morphants (n = 30–45) were *iv* infected with S *Mabs* (tdTomato, ≈150 CFU). Kinetic of granuloma formation. Graphs represent the mean ± SEM from three independent experiments. Significance was assessed by Fisher’s exact test of a contingency table.
(TIF)

S1 Movie Time-lapse microscopy of neutrophils mobilization in the muscle following *Mabs* injection. 72hpf *Tg(mpx*:*eGFP)* larvae were intramuscularly infected with ≈100 *Mabs* R expressing tdTomato and live imaged by confocal fluorescence microscopy, every 10 min from 10 mpi to 150 mpi. Neutrophils are rapidly recruited to the intramuscularly injected bacteria and efficiently phagocytose *Mabs*.
(MP4)

S2 Movie Three-dimensional reconstruction of neutrophils recruitment to R-cord in WT embryos. *Tg(mpx*:*eGFP)* larvae were *iv* infected with ≈100 *Mabs* R expressing tdTomato and imaged by confocal fluorescence microscopy at 3 dpi. Neutrophils are efficiently recruited to the R-cord in WT context.
(MP4)

S3 Movie Three-dimensional reconstruction of neutrophils recruitment to R-cord in *tnfr1* morphant embryos. *Tg(mpx*:*eGFP)* larvae were *iv* infected with ≈100 *Mabs* R expressing tdTomato and imaged by confocal fluorescence microscopy at 3 dpi. Neutrophils failed to be recruited to the R-cord in absence of TNFR1.
(MP4)

S4 Movie Three-dimensional reconstruction of neutrophils recruitment to R-abscess in WT embryos. *Tg(mpx*:*eGFP)* larvae were *iv* infected with ≈100 *Mabs* R expressing tdTomato and imaged by confocal fluorescence microscopy at 5 dpi. Neutrophils are efficiently recruited to the R-abscess in WT context.
(MP4)

S5 Movie Three-dimensional reconstruction of neutrophils recruitment to R-abscess in *tnfr1* morphant embryos. *Tg(mpx*:*eGFP)* larvae were *iv* infected with ≈100 *Mabs* R expressing tdTomato and imaged by confocal fluorescence microscopy at 5 dpi. Neutrophils failed to be recruited to the R-abscess in absence of TNFR.
(MP4)

S6 Movie Three-dimensional reconstruction of a *Mabs* granuloma and associated neutrophils behavior in WT embryos. *Tg(mpx*:*eGFP)* larvae were *iv* infected with ≈100 *Mabs* R expressing tdTomato and imaged by confocal fluorescence microscopy at 3 dpi. WT-granuloma harbors numerous neutrophils.
(MP4)

S7 Movie Three-dimensional reconstruction of a *Mabs* granuloma and associated neutrophils behavior in *tnfr1* morphant embryos. *Tg(mpx*:*eGFP) tnfr* morphants were *iv* infected with ≈100 *Mabs* R expressing tdTomato and imaged by confocal fluorescence microscopy at 3 dpi. The granuloma is profoundly depleted of neutrophil.
(MP4)
