# Supplementary figures and images for: Mycobacterium abscessus-Induced Granuloma Formation Is Strictly Dependent on TNF Signaling and Neutrophil Trafficking
Source: PLoS Pathog. 2016 Nov 2;12(11):e1005986. doi: 10.1371/journal.ppat.1005986 (PMC5091842; doi:10.1371/journal.ppat.1005986)

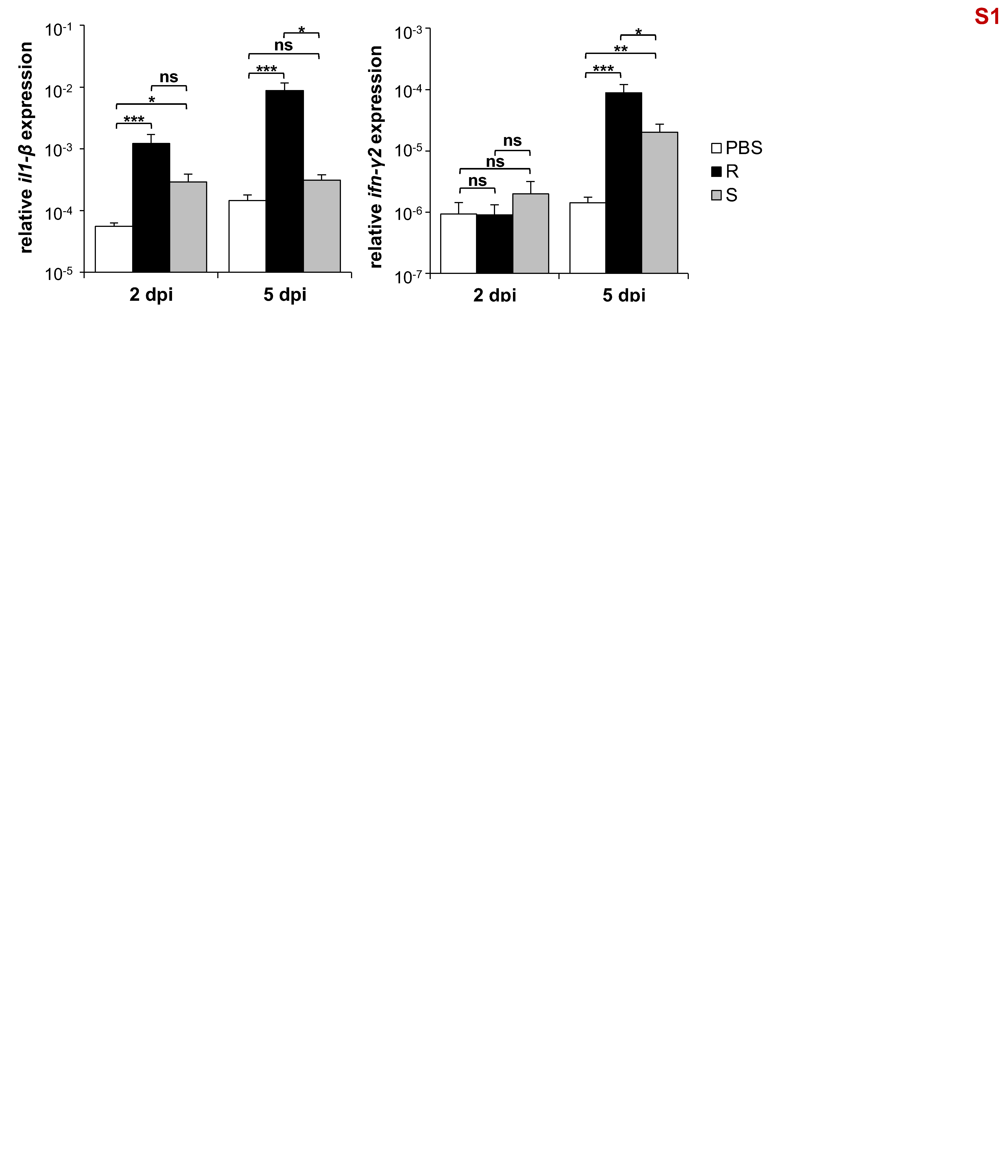

Supplement: S1 Fig — IL-1β and IFN-γ2 pro-inflammatory cytokines response during Mabs infection. PBS, ≈150 Mabs R or S variants were iv injected. Quantitative RT-PCR using ef1a as a reference gene was performed to measure the relative expression of these cytokines in whole embryo assessed at 2 and 5 dpi. Results are presented as mean log10 ± SEM of three independent experiments and statistical significance was determined by Kruskal-Wallis test with Dunns post-test. (TIF) [file ppat.1005986.s001.tif]

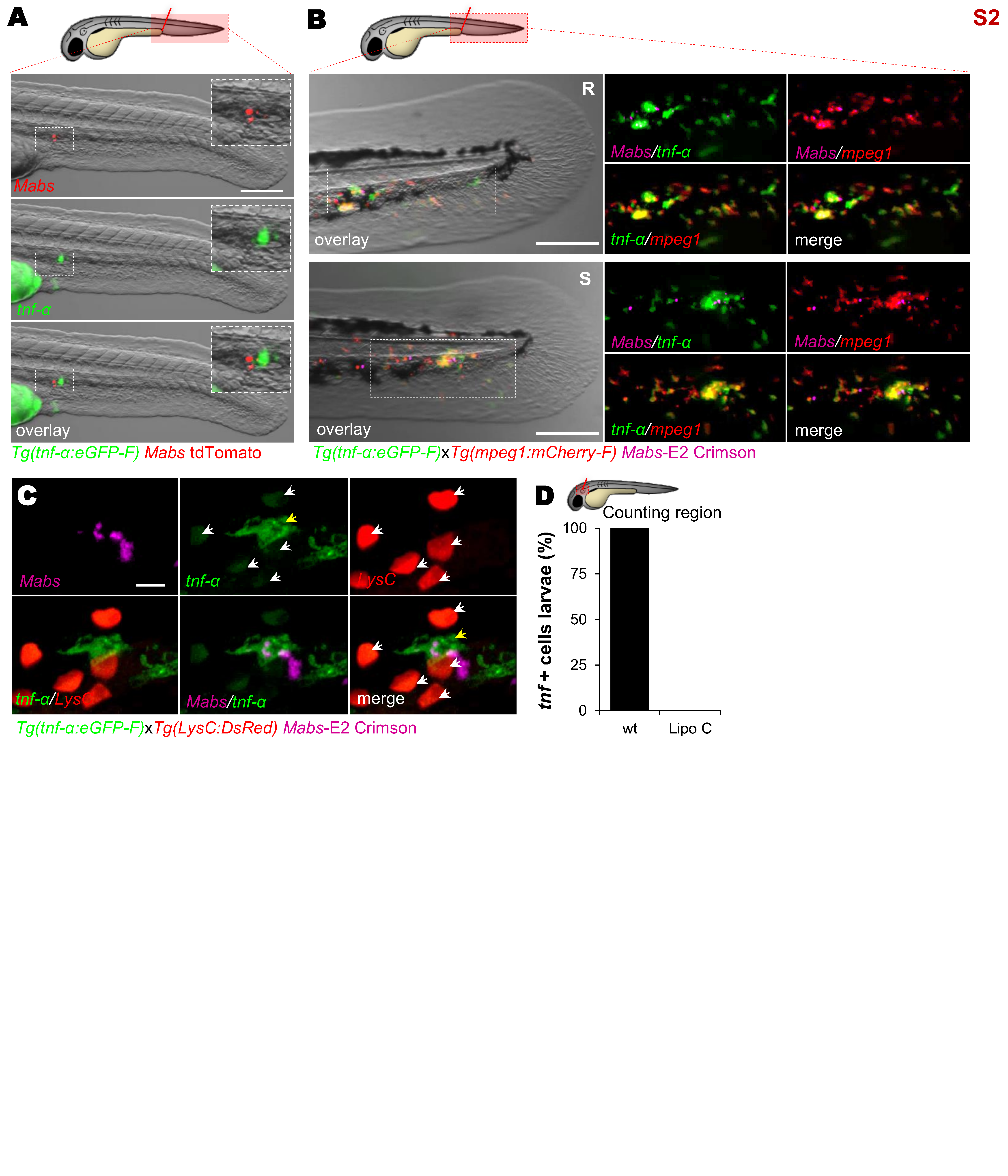

Supplement: S2 Fig — (A) Tg(tnf-α:eGFP-F) embryos were infected into caudal vein with ≈100 Mabs-expressing tdTomato. Microscopy showing the representative expression of tnf-α close to the injection site at 2 hpi. Scale bar, 300 μm. (B-C) Fluorescence microscopy analysis of the GFP expression in Tg(tnf-α:eGFP-F/mpeg1:mCherryF) (B) or Tg(tnf-α:eGFP-F/LysC:DsRed) (C) double transgenic embryos at 1 day following intravenous infection by either ≈100 R- or S-expressing E2-Crimson. (B) Close-up to the tip of the tail revealing that the transcriptomic tnf-α expression is detected in infected macrophages or in macrophages close to the infected tissue. Scale bars, 200 μm. (C) Confocal images of a single Mabs-infected macrophage (yellow arrow) close to the injection site induced a strong tnf-α expression. Neutrophils are indicated with white arrows. Scale bar, 10 μm. (D) tnf-α expression was tested in Tg(tnf-α:GFP-F) zebrafish embryo in absence of macrophages (lipo-clodronate injection). Embryos were infected with Mabs (R variant, ≈100 CFU) in the otic cavity and the proportion of infected embryos with eGFP-positive cells at 2 hpi counted. Graphs represent the mean value of two independent experiments (n = 10). (TIF) [file ppat.1005986.s002.tif]

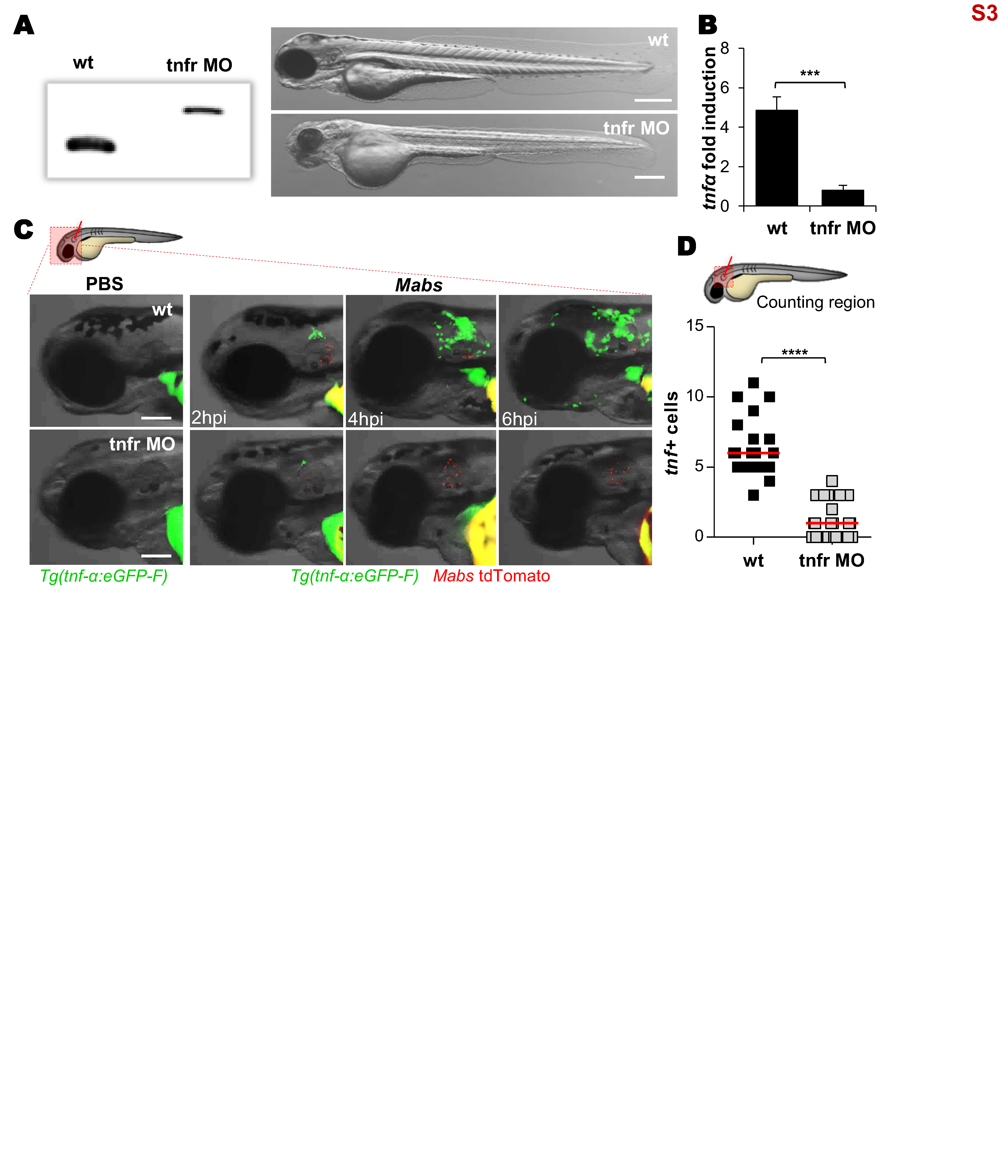

Supplement: S3 Fig — (A-D) Efficiency of morpholino against zebrafish TNFR1. (A) Injection of splice-site blocking antisens morpholinos targeting the TNF receptor 1 (tnfr) leads to a total absence of native tnfr1 transcript. Comparison between WT embryos and tnfr morphants transcripts (2 dpf) reveals the complete absence of native transcript. Bright-field microscopy image comparing the whole morphology appearance of WT embryos versus tnfr morphants at 2 dpf, showing that morpholino knockdown injection produces a moderate hypomorph phenotype: short body length, big yolk sac, smaller swim bladder, small eyes, reduced pigmentation, hindbrain defects, somites poorly organized and epidermic alterations. Scale bars, 200 μm. (B-D) To check the effect of tnfr1 loss-of-function on the tnf-α production, PBS or tdTomato-Mabs (R variant, ≈100 CFU) were injected intravenously (B) or into the otic cavity of either WT or tnfr morphants Tg(tnf-α:eGFP-F) larvae (C-D). (B) qRT-PCR of tnfα (normalized to ef1α) upon Mabs infection. Fold induction compared to entire PBS- injected fishes at 3 dpi. Error bars indicate SEM. (C) Bright-field and fluorescence overlay microscopy showing the real-time visualization of the transcriptional tnf-α expression close to the injection site assessed at 2, 4 and 6 hpi. Scale bars, 100 μm. (D) Number of tnf-α positive cells per infected larvae evaluated at 2 hpi using confocal microscopy. Each symbol represents individual embryos and horizontal lines indicate the median values. (B and D) Statistical significance was assessed by one-tailed Mann-Whitney’s t test. TNF-α expression subsequent to the infection is impaired in tnfr morphants. Results are presented as average number from two experiments. (TIF) [file ppat.1005986.s003.tif]

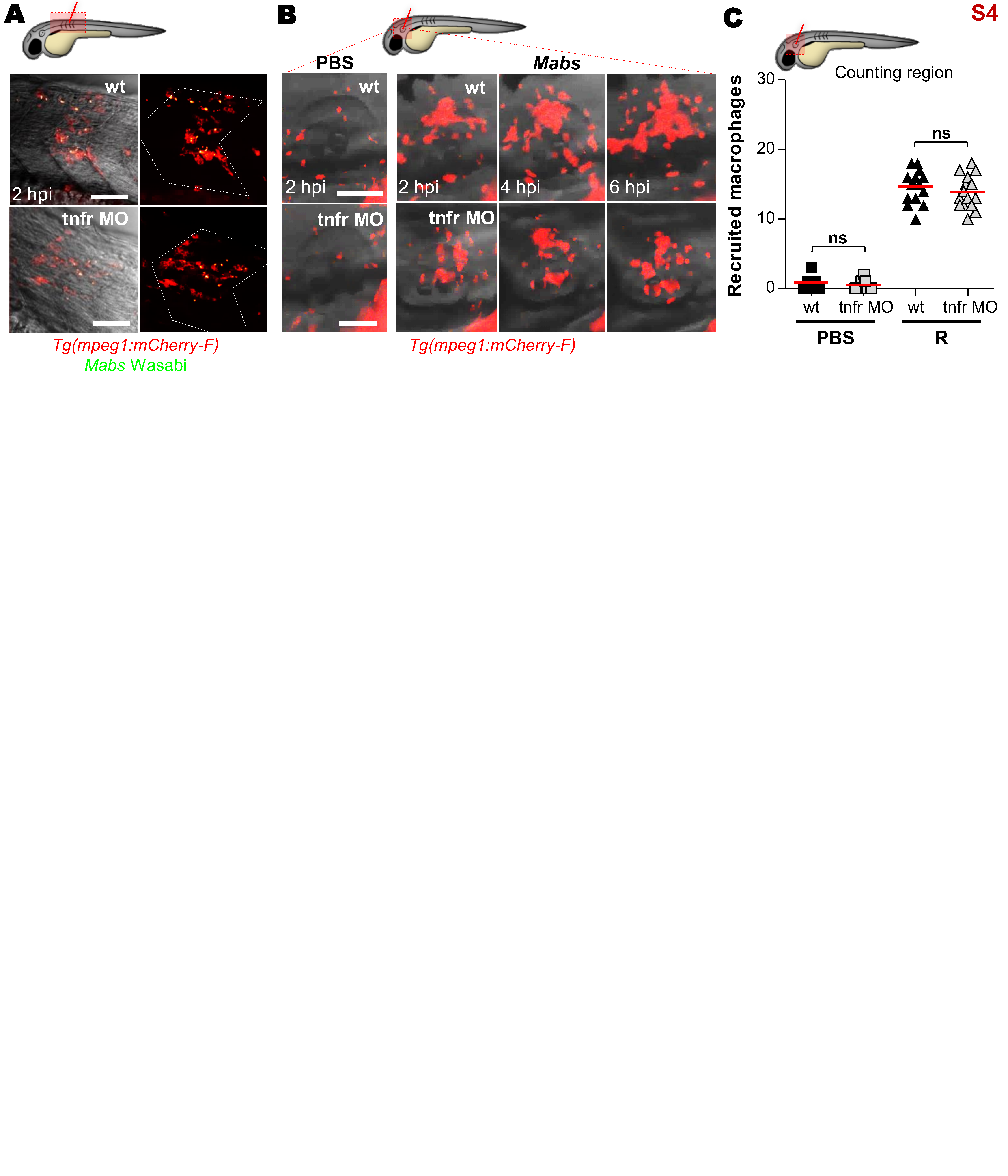

Supplement: S4 Fig — To evaluate the effect of absence of TNF signaling on early and late macrophages recruitment, WT or tnfr morphants Tg(mpeg1:mCherry-F) larvae were injected with either PBS or Wasabi-expressing Mabs (R variant) into the muscle (A) or otic cavity (B-C), monitored and imaged using confocal microscopy to measure macrophage recruitment. (A) Representative confocal microscopy of macrophages recruitment into the infected muscle at 2 hpi (dotted line outlines 2 somitic muscles). Scale bars, 100 μm. (B-C) Dynamic of macrophage recruitment at the infection site assessed at 2, 4 and 6 hpi (Scale bars, 50 μm) (B) and number of recruited macrophages at 2 hpi (C). Results are presented as average number from two experiments. Each symbol represents individual embryos and horizontal lines indicate mean values. Significance was assessed by one-tailed unpaired Student’s t test. In both WT- and tnfr morphant-infected animals, macrophages are recruited towards bacteria at the same rate at early time post-infection. However, while the number of newly recruited macrophages increased progressively in WT larvae from 2 hpi to 6 hpi, the number of recruited macrophages remains constant in tnfr morphants. (TIF) [file ppat.1005986.s004.tif]

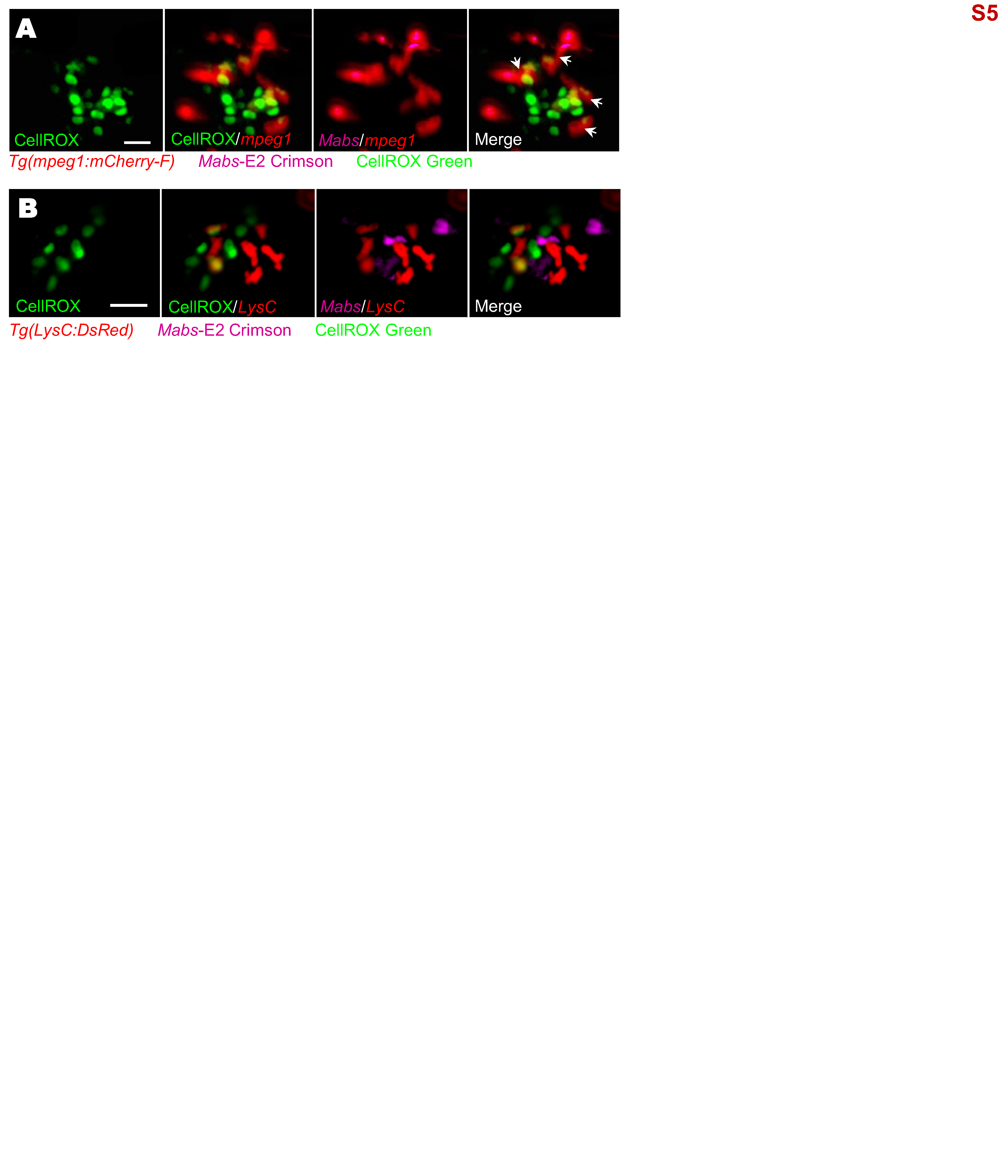

Supplement: S5 Fig — Evaluation of ROS induction in infected embryos using CellROX Green fluorescent. Tg(mpeg1:mCherry-F) (A) or Tg(LysC:DsRed) (B) were iv infected with E2 Crimson-expressing Mabs (R variant) and monitored for the ROS detection. (A) Confocal microscopy of agglomerates of hematopoietic cells display ROS production. Scale bar, 15 μm. Arrows indicate ROS-positive macrophages. (B) Mobilization of neutrophils close to infected tissue with agglomerates of hematopoietic cells producing ROS is revealed by confocal microscopy. Scale bar, 20 μm. (TIF) [file ppat.1005986.s005.tif]

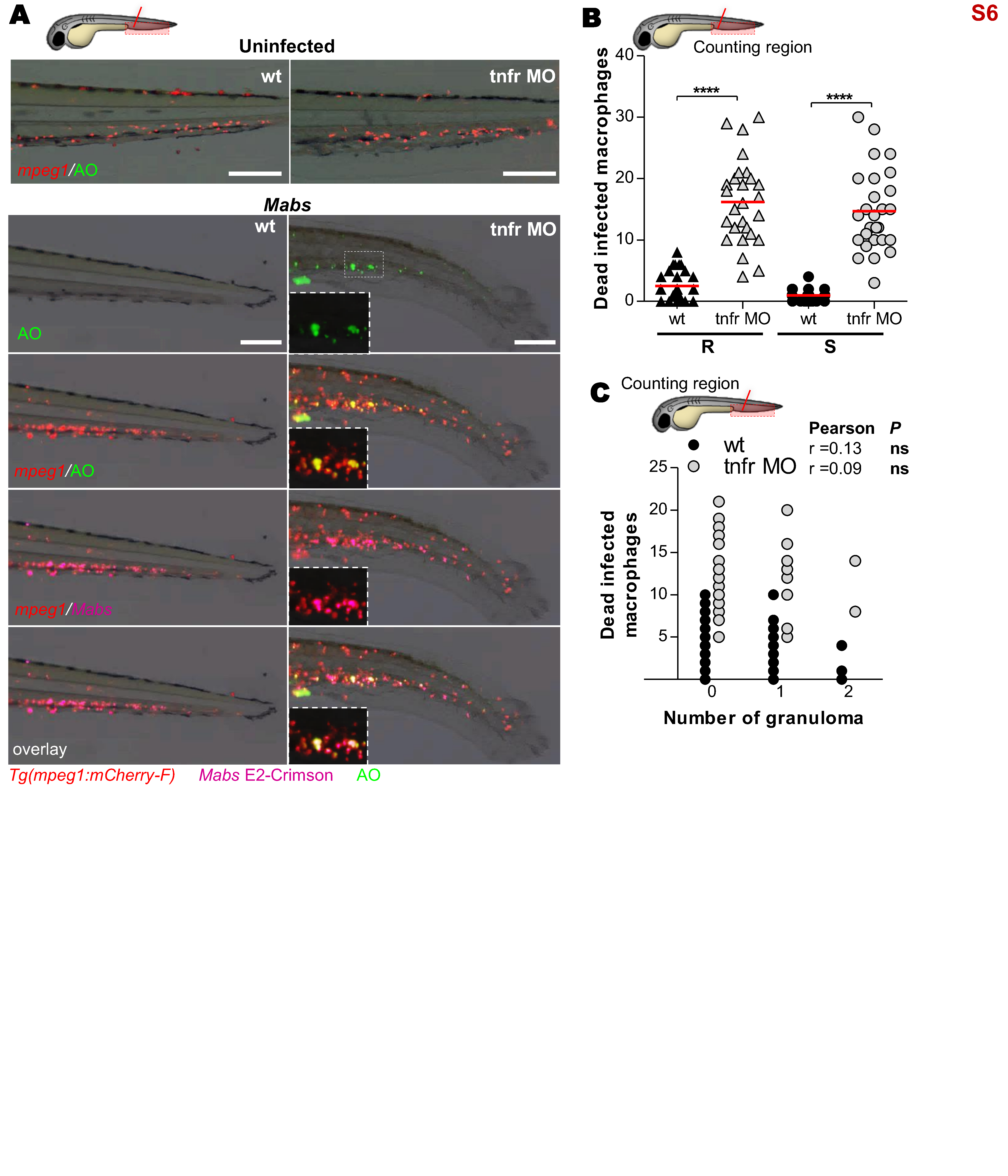

Supplement: S6 Fig — (A and B) WT or tnfr morphants Tg(mpeg1:mCherry-F) were iv infected with either R or S variants of Mabs expressing E2 Crimson (≈150 CFU) and stained for dead macrophages with acridine orange (AO). (A) Representative microscopy showing the dead macrophages in 2 dpi R-infected embryos. Scale bars, 80 μm. (B) Number of dead infected macrophages evaluated using confocal microscopy at 2 dpi. Each symbol represents individual embryos and horizontal lines indicate mean values. (C) WT, il8 or csf3r morphants Tg(mpeg1:mCherry-F) were iv infected with either R or S variants (≈150 CFU) and stained for dead macrophages with acridine orange (AO). Statistical significance was determined by one-tailed Student’s t test (B) or Pearson correlation (C). Results are presented as the average number from two experiments. (TIF) [file ppat.1005986.s006.tif]

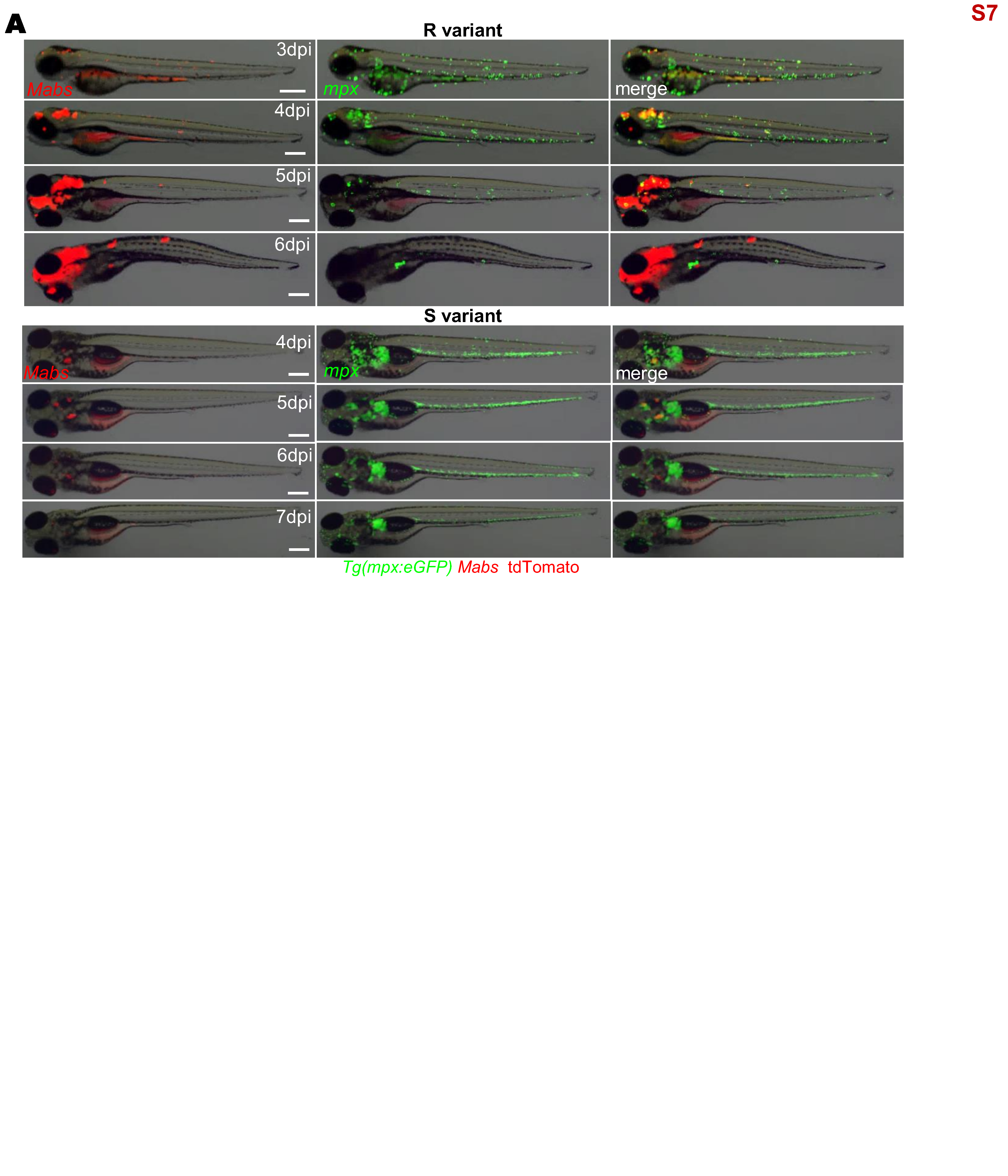

Supplement: S7 Fig — To investigate the behavior of neutrophils following the systemic Mabs infection, either R- or S-tdTomato (≈150 CFU) were iv injected in Tg(mpx:eGFP) embryo. Infected embryos were monitored and imaged at different time points following injection to monitor neutrophils recruitment at the infection foci. Bright-field and fluorescence overlay image showing representative recruitment of neutrophils following infections. Scale bars, 200 μm. (TIF) [file ppat.1005986.s007.tif]

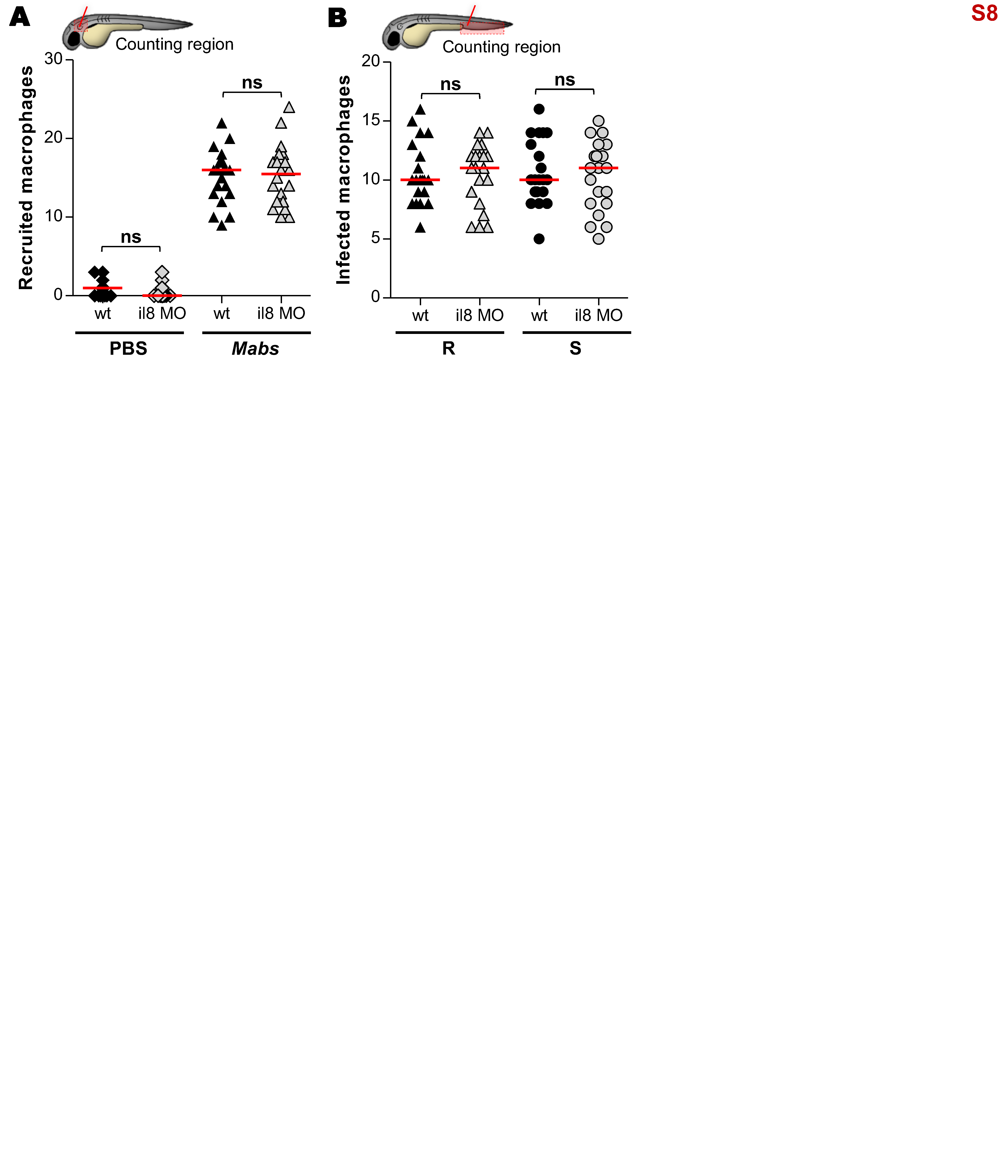

Supplement: S8 Fig — WT or il8 morphants Tg(mpeg1:mCherry-F) larvae were injected with either PBS or R- or S-tdTomato (≈150 CFU) into the otic cavity (A) or the caudal vein (B) and monitored for macrophage recruitment and phagocytosis using confocal microscopy. (A) Number of recruited macrophages into the otic cavity at 2 hpi. (B) Number of infected macrophages in the CHT at 4 hpi. (A-B) Significance was assessed by one-tailed unpaired Student’s. Results are presented as average number from two experiments. Each symbol represents individual embryos and horizontal lines indicate mean values. (TIF) [file ppat.1005986.s008.tif]

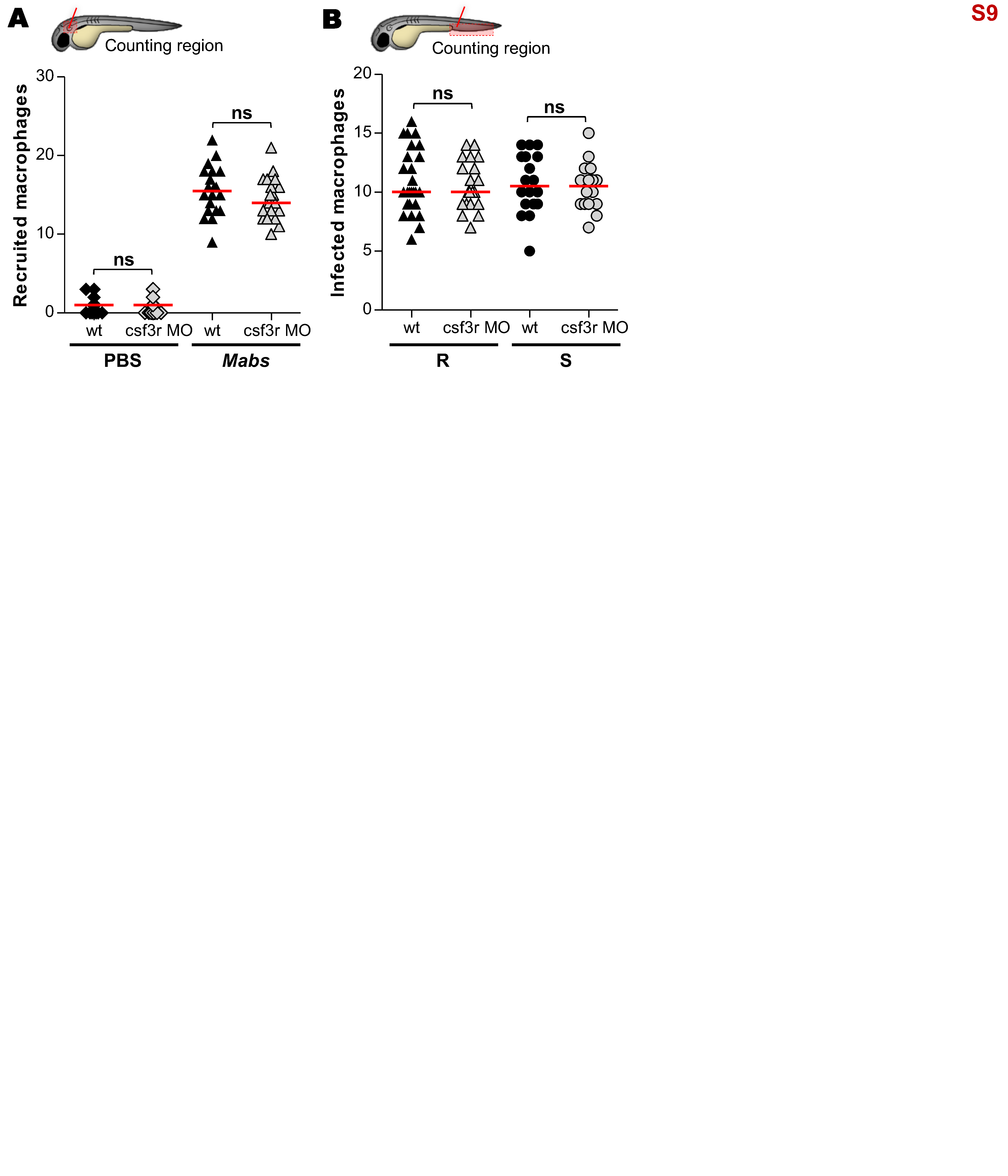

Supplement: S9 Fig — WT or csf3r morphants Tg(mpeg1:mCherry-F) larvae were injected with either PBS or R- or S-tdTomato (≈150 CFU) into the otic cavity (A) or the caudal vein (B) and monitored for macrophage recruitment and phagocytosis using confocal microscopy. (A) Mean number of recruited macrophages into the otic cavity at 2 hpi. (B) Mean number of infected macrophages in the CHT at 4 hpi. (A-B) Significance was assessed by one-tailed unpaired Student’s. Results are presented as average number from two experiments. Each symbol represents individual embryos and horizontal lines indicate mean values. (TIF) [file ppat.1005986.s009.tif]

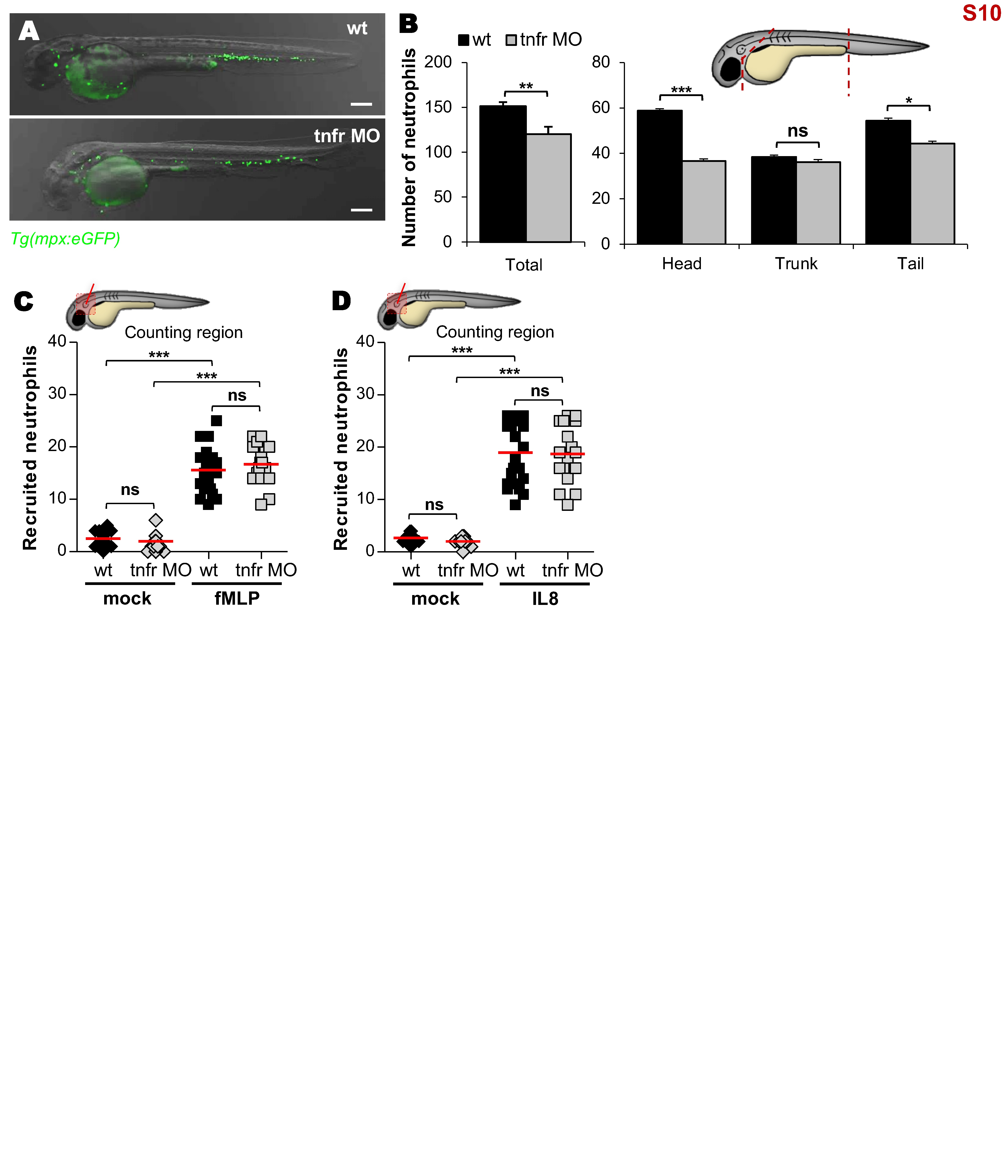

Supplement: S10 Fig — (A) Representative bright-field and fluorescence overlay image of WT versus tnfr morphants Tg(mpx:eGFP) embryos at 2 dpf. Scale bars, 200 μm. (B) Quantification of basal number of neutrophils in whole (left) or detailed in the head, in the trunk and tail (right) of 2 dpf embryos (n = 12). Graphs represent the mean ± SEM. (C and D) Mean number of recruited neutrophils into the otic cavity in response to mock, fMLP (C) or IL8 (D) injection in WT and tnfr morphants Tg(mpx:eGFP) embryos monitored at 3 hpi. Each symbol represents an individual embryo and horizontal lines indicate the mean values. Significance was assessed by one-tailed unpaired Student’s t test comparing both embryos per category (B) or by ANOVA with Tukey post-test (C and D). (B-D) Results are representative of two independent experiments. (TIF) [file ppat.1005986.s010.tif]

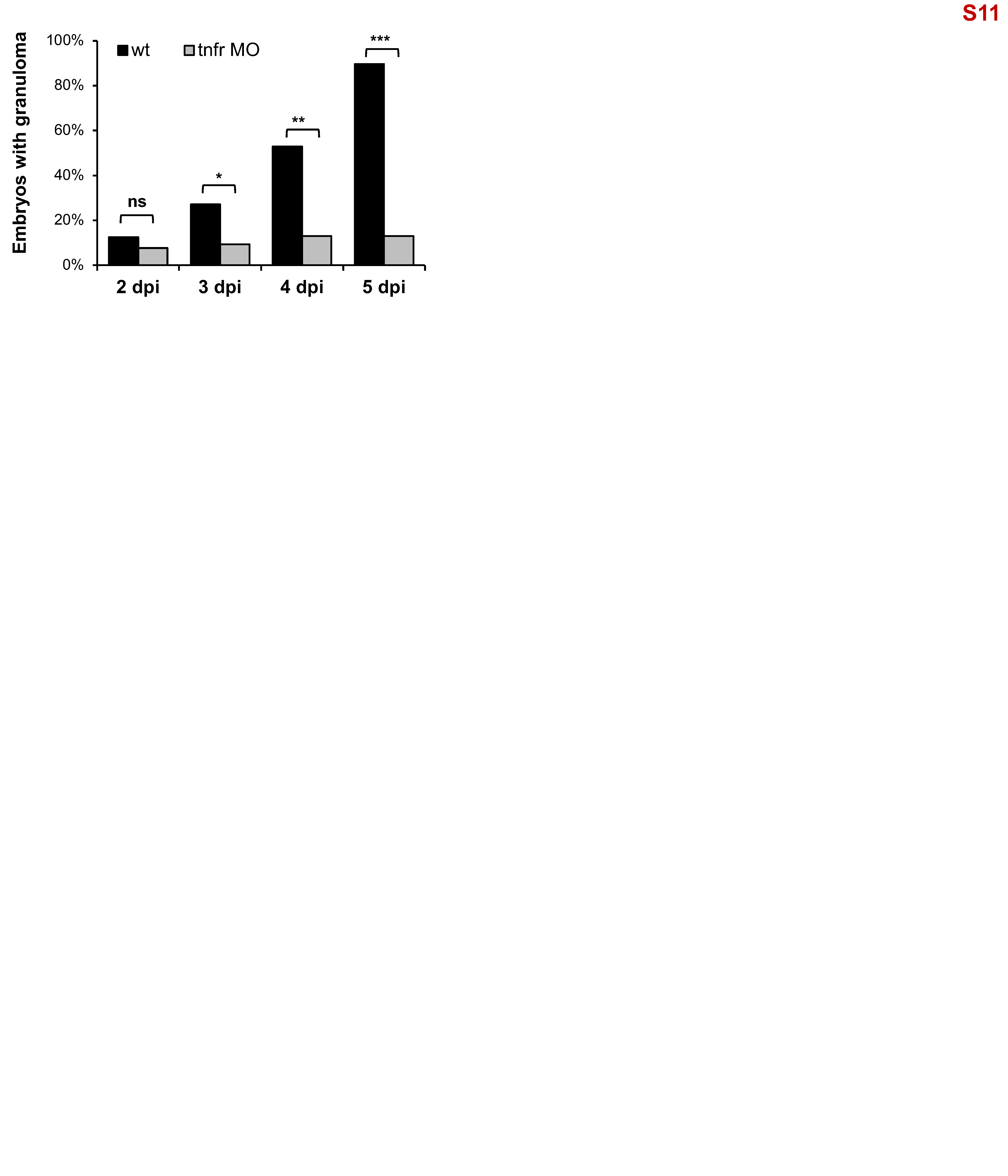

Supplement: S11 Fig — WT or tnfr morphants (n = 30–45) were iv infected with S Mabs (tdTomato, ≈150 CFU). Kinetic of granuloma formation. Graphs represent the mean ± SEM from three independent experiments. Significance was assessed by Fisher’s exact test of a contingency table. (TIF) [file ppat.1005986.s011.tif]
